# High-Speed, Pixel-Super-resolved Compressive Second Near-Infrared Fluorescence In Vivo Imaging

**DOI:** 10.34133/research.1146

**Published:** 2026-03-19

**Authors:** Zhen Pan, Dalong Qi, Hongxin Zhang, Jiali Yao, Long Cheng, Ning Xu, Chengyu Zhou, Wenzhang Lin, Hongmei Ma, Yunhua Yao, Yuecheng Shen, Lianzhong Deng, Fan Zhang, Zhenrong Sun, Shian Zhang

**Affiliations:** ^1^ State Key Laboratory of Precision Spectroscopy, School of Physics, East China Normal University, Shanghai 200062, China.; ^2^ Laboratory of Advanced Materials, Department of Chemistry, College of Smart Materials and Future Energy, State Key Laboratory of Molecular Engineering of Polymers, Shanghai Key Laboratory of Molecular Catalysis and Innovative Materials, Fudan University, Shanghai 200433, China.; ^3^ College of Science, Shanghai Institute of Technology, Shanghai 201418, China.; ^4^ Collaborative Innovation Center of Extreme Optics, Shanxi University, Taiyuan 030006, China.

## Abstract

Conventional second near-infrared (NIR-II; 1,000 to 1,700 nm) fluorescence imaging cannot simultaneously achieve a high signal-to-noise ratio and motion-artifact-free capture of rapid physiological dynamics. Here, we introduce NIR-II compressive fluorescence imaging (COFI), a high-speed, pixel-super-resolved compressive imaging technique that encodes dynamics into single frames using a high-speed spatial light modulator and a low-frame-rate NIR-II camera. A hybrid reconstruction algorithm integrating a denoising convolutional neural network with an enhanced super-resolution generative adversarial network subsequently restores high-fidelity videos. The system achieves 3.3 kiloframes per second with a space–bandwidth–time product of 4.22 × 10^8^ pixels/s without compromising intrinsic sensitivity. Compared to conventional short-exposure imaging with the same duration of 500 μs, NIR-II COFI achieves a 36% improvement in signal-to-noise ratio. Furthermore, using bright 1,525-nm nanoparticle probes, we demonstrate multicomponent phosphorescence lifetime imaging, high-speed motion tracking, and real-time visualization of murine intestinal peristalsis in both awake and anesthetized states. This work facilitates deep-tissue, high-speed in vivo imaging of fast biological processes.

## Introduction

Second near-infrared (NIR-II; 1,000 to 1,700 nm) fluorescence imaging has rapidly evolved into a transformative tool in biomedical imaging, owing to its superior optical characteristics [1–8]. Compared to conventional fluorescence imaging in the visible (400 to 700 nm) and first near-infrared (700 to 900 nm) ranges, the NIR-II window substantially reduces photon scattering by 3 to 5 orders of magnitude, minimizes tissue autofluorescence, and lowers the optical absorption coefficient of biological tissues by more than 80% [9–14]. These advantages enable millimeter-scale penetration while preserving micrometer-level spatial resolution [15]. Moreover, the substantially lower photon energy (approximately one-third that of visible light) reduces phototoxicity and photobleaching, thereby extending the feasible duration for in vivo dynamic monitoring to several hours [16]. Consequently, NIR-II fluorescence imaging has become instrumental in biomedical applications, such as tumor therapeutics [17], cerebrovascular function assessment [18], and neuroimaging of the brain [19]. Therefore, advancing NIR-II fluorescence imaging technologies is critical for pushing the boundaries of biomedical imaging and enhancing diagnostic and therapeutic capabilities [20].

Despite its widespread adoption, NIR-II fluorescence imaging continues to face challenges in achieving a higher spatiotemporal resolution for complex in vivo applications. Since Welsher et al.’s groundbreaking demonstration of NIR-II fluorescence in vivo imaging in 2009 [21], researchers in this field have pioneered a suite of innovative probes [22–24]—including quantum dots [25], rare-earth-doped nanoparticles [26], and organic small-molecule dyes [27], which have substantially expanded the range of NIR-II applications. Concurrently, integrations with confocal microscopy [28], light-sheet illumination [29], and super-resolved structured illumination microscopy [30] have progressively enhanced the spatial resolution of NIR-II fluorescence imaging. For example, Zubkovs et al. [31] reported 3-dimensional (3D) chloroplast imaging at 20 frames per second (fps) with a sub-1.5-μm section thickness using an NIR-II spinning-disc confocal microscope in 2018, achieving a spatial resolution smaller than 600 nm. Wang et al. [30] employed the NIR-II structured illumination microscopy technique combined with digitally scanned laser beam-generated structured illumination patterns to achieve a volumetric resolution of 1.7 × 1.1 × 1.6 μm and complete 3D imaging with single-image exposure time durations of 100 to 150 ms. By integrating NIR-II fluorescence microscopy with clinically approved indocyanine green-labeled red blood cells and a high-speed NIR-II camera, Zhang et al. [32] achieved 350-μm-deep spinal cord vascular imaging and 100-fps dynamic tracking of red blood cell velocity in 2022. Although NIR-II fluorescence microscopy has achieved super-resolution performance beyond the classical diffraction limit through advanced optical and computational methods [28–32], its imaging speed remains constrained by the slow response of cooled NIR-II detectors [33] and the reliance on scanning-based acquisition modes. Consequently, NIR-II fluorescence imaging faces a trade-off between achieving kiloframes per second (kfps) rates and maintaining the signal-to-noise ratio (SNR) for high-speed in vivo imaging, thereby falling short of capturing rapid physiological processes such as neural activities and hemodynamic changes [20]. To date, few studies have tackled the speed bottleneck of NIR-II fluorescence imaging through innovations in optical system design. This technological imbalance underscores the limitations of conventional imaging paradigms in photon flux utilization and multidimensional data acquisition, emphasizing the urgent need for synergistic advancements in computational imaging algorithms and next-generation infrared detector technologies.

To overcome the limitations of NIR-II imaging techniques imposed by InGaAs detectors, we developed a high-speed, pixel-super-resolved compressive NIR-II fluorescence imaging technique (NIR-II compressive fluorescence imaging [COFI]). This system integrates a high-speed programmable spatial light modulator (SLM) with an InGaAs camera, enabling the acquisition of spatiotemporal information from dynamic scenes in a single exposure. As a result, the frame rate of NIR-II COFI achieves more than an order-of-magnitude increase compared to the native frame rate of the system’s camera. Additionally, to ensure high-fidelity recovery of compressed scenes, we designed a hybrid spatiotemporal reconstruction framework combining the alternating direction method of multipliers (ADMM) and a denoising convolutional neural network with a PhysicsUpdate module (DPNet). Furthermore, pixel super-resolution (PSR) was introduced via an enhanced super-resolution generative adversarial network (ESRGAN) to overcome hardware resolution limits and achieve detailed image reconstruction. This algorithm is referred to as DPNet-ESRGAN. Experimentally, the system achieved an imaging speed of 3.3 kfps, which improves the NIR-II detector’s imaging speed by 20 times without compromising the detector’s intrinsic sensitivity, thereby overcoming the inherent limitations of conventional NIR-II fluorescence imaging. With an imaging field of view (FOV) of 2.4 mm × 1.92 mm, the NIR-II COFI system attains a spatial resolution of 16.45 lp/mm, corresponding to a space–bandwidth–time product (SBTP), defined as the effective number of resolved pixels multiplied by the frame rate, of 4.22 × 10^8^ pixels/s. By contrast, the most widely used high-speed NIR-II cameras under comparable cooling conditions can achieve a maximum SBTP of only 3.6 × 10^7^ pixels/s [34], while high-end systems achieving comparable SBTPs are often prohibitively expensive, such as the C-Red One camera. Additionally, we compared the imaging parameters between the NIR-II COFI system and representative state-of-the-art NIR-II imaging techniques in Note S1 to more clearly demonstrate the advantages of the developed system. Using NIR-II COFI, we successfully performed phosphorescence lifetime imaging of multicomponent rare-earth-doped nanoparticle samples by exploiting its flexibility in temporal encoding and demonstrated its superiority in maintaining a sufficient SNR with motion imaging of fluorescent microspheres. Furthermore, we applied it in in vivo imaging and realized a visualization of the dynamic process of murine intestinal peristalsis in anesthetized and awake states, which is difficult to distinguish with traditional low-frame-rate NIR-II imaging methods. The results demonstrate the considerable potential of NIR-II COFI in biomedical research, offering an accurate and efficient approach for studying complex biological dynamic processes and laying the foundation for future deep-tissue imaging and in vivo biological research.

## Results

### Characterization of NIR-II COFI system

**Fig. 1.**
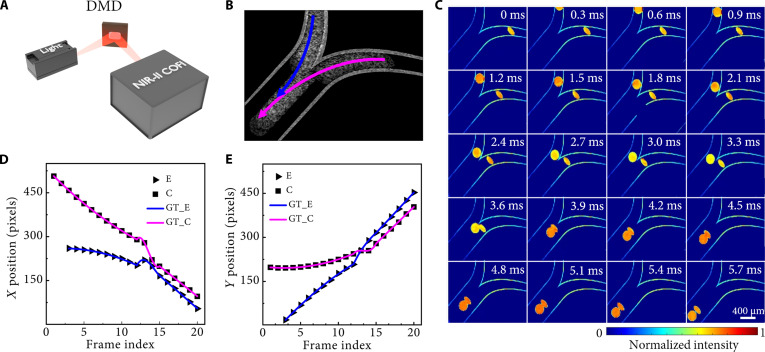
Validation of high-speed tracking capability using second near-infrared (NIR-II) compressive fluorescence imaging (COFI). (A) Schematic of the experimental setup combining digital micromirror device (DMD)-based spatial encoding with NIR-II detection. (B) Conventional time-integrated image (6-ms exposure) showing blurred motion trajectories (indicated by arrows). (C) Reconstructed frames from a snapshot with the NIR-II COFI system. (D and E) Comparisons of the centroid positions of the 2 cell patterns with different shapes in reconstruction to the ground truth (GT) in the *x* and *y* directions, respectively. E, elliptical cell; C, circular cell.

To evaluate the capability of the NIR-II COFI system in tracking rapidly moving targets, a dynamic scene mimicking cell movement with various shapes and velocities was captured. As shown in Fig. 1A, a halogen light source filtered through a 1,550-nm band-pass filter (10-nm bandwidth) provided NIR-II illumination to the dynamic scene, which was generated by a digital micromirror device (DMD; DLP6500) refreshing pregenerated images at 3.3 kHz. The NIR-II COFI system was synchronized with the DMD to acquire the dynamic sequence. Figure 1B shows the spatiotemporal integration image of the dynamic scene acquired by the system with an exposure time of 6 ms, where blue and pink arrows denote the predefined motion trajectories of circular and elliptical cells, respectively. Figure 1C presents the reconstructed 20-frame sequence (300-μs interframe interval) with the DPNet-ESRGAN algorithm, revealing the spatiotemporal evolution of object morphology and motion trajectories that were obscured in the integrated image. Quantitative assessment was performed by tracking the centroid coordinates (*x*–*y*) of elliptical and circular cells across the reconstructed frames, and the results are shown in Fig. 1D and E. It is evident that the temporal trajectories of the centroid coordinates reconstructed by the proposed algorithm exhibit high consistency with the ground-truth references for both cell shapes. These results validate the high measurement accuracy of the system at an ultrahigh frame rate of 3.3 kfps. It is worth noting that a compression ratio of 20 indicates that we can improve the imaging speed of the camera by more than an order of magnitude.

**Fig. 2.**
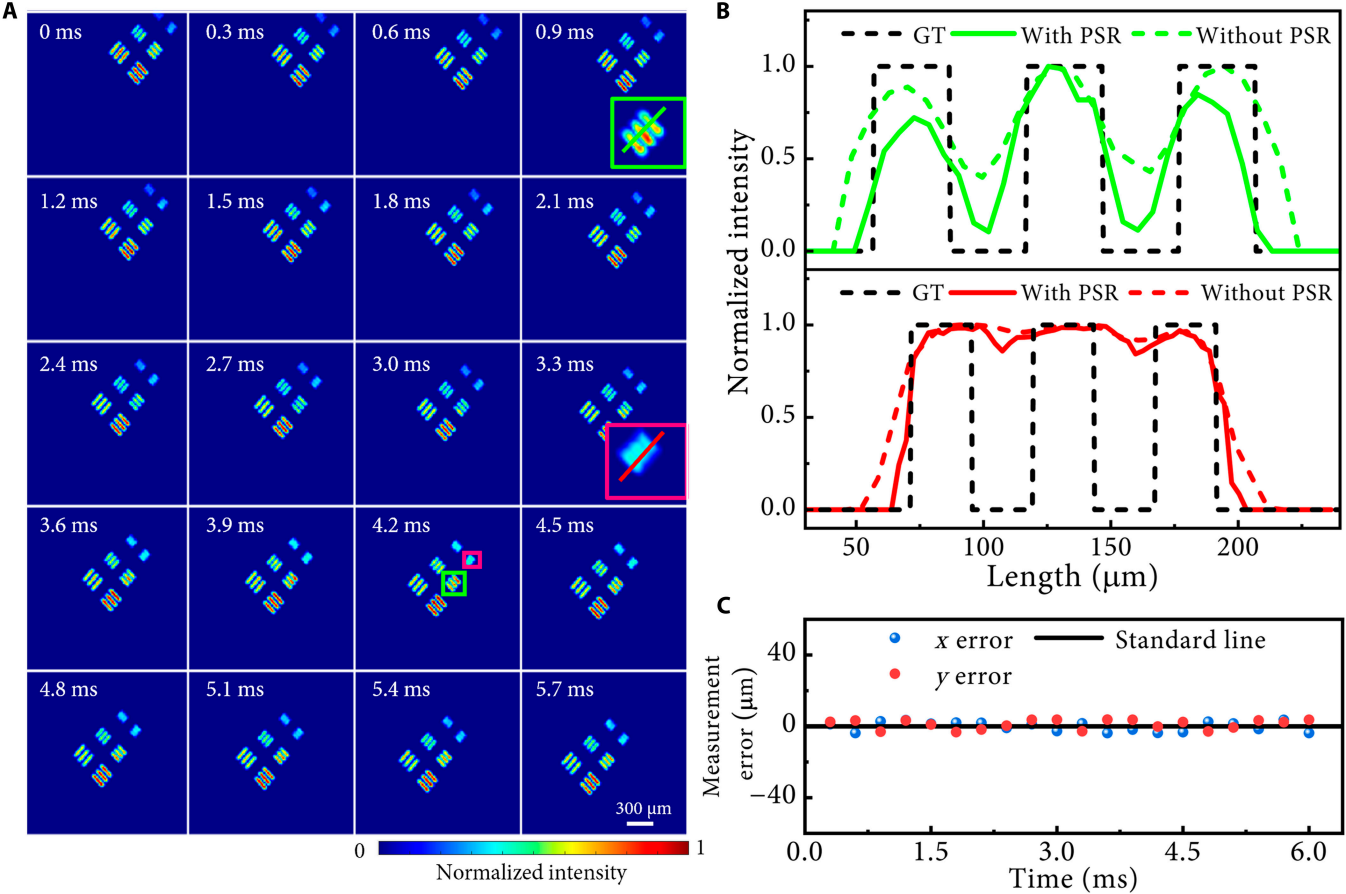
Spatial resolution improvement of the NIR-II COFI system with the DPNet-enhanced super-resolution generative adversarial network (ESRGAN) algorithm. (A) Reconstructed frames of a moving resolution test target. The regions marked with green and red boxes are magnified and displayed in frames at 0.9 and 3.3 ms, respectively. (B) Intensity profiles along the green and red solid lines within the magnified boxes in (A) with and without pixel super-resolution (PSR). (C) Measurement errors along the *x*- and *y*-axes of the reconstructed image from the ideal resolution test pattern at each time instant.

To validate the spatial resolution enhancement achieved by the DPNet-ESRGAN algorithm in the NIR-II COFI system, a dynamic resolution test pattern was projected by a DMD for evaluation. Following a series of tests, we selected 3 optimal line-pair sizes generated by the DMD—45.6, 60.8, and 76 μm (line center to line center). The 3 sets of resolution test bars, oriented at 45° to the horizontal direction, were projected and translated at a constant velocity diagonally down to the left. Figure 2A illustrates the reconstruction results obtained using the DPNet-ESRGAN algorithm. Moreover, a comparative analysis of conventional plug-and-play ADMM (PnP-ADMM) [35] and the proposed algorithm (see details in Note S2) indicates that the latter provides a markedly enhanced spatial resolution. To quantitatively assess the resolution improvement of the PSR module in our algorithm, Fig. 2B presents the ideal and reconstructed intensity profiles of the smallest and intermediate bar pairs in the frame at 4.2 ms. The black dashed lines denote the ground-truth cross-sectional intensity profiles, while the green (red) solid and dashed lines represent profiles reconstructed by the proposed algorithm with and without the ESRGAN module, respectively. Evidently, integration of the super-resolution module leads to substantial improvements in both detail preservation and spatial resolution in the reconstructed outputs. Notably, the smallest resolvable bar pair corresponds to a measured system resolution of 16.45 lp/mm (lower subplot, Fig. 2B), which is comparable to the native resolution achieved by direct, uncompressed camera imaging. Temporal stability was quantified by computing positional errors along the *x*- and *y*-axes between reconstructed frames and ground-truth patterns over time, as shown in Fig. 2C. The positional errors were obtained by subtracting each test line pair’s preset centroid from the measured centroid and averaging, yielding maximum positional inaccuracies below 3.8 μm, indicating the stable imaging and high accuracy of the NIR-II COFI system. With a 2.4 mm × 1.92 mm FOV, the SBTP of the system reaches 4.22 × 10^8^ pixels/s, enabling over an order-of-magnitude improvement in information acquisition efficiency compared to conventional NIR-II fluorescence imaging systems.

### Phosphorescence lifetime imaging of multicomponent nanoparticles

**Fig. 3.**
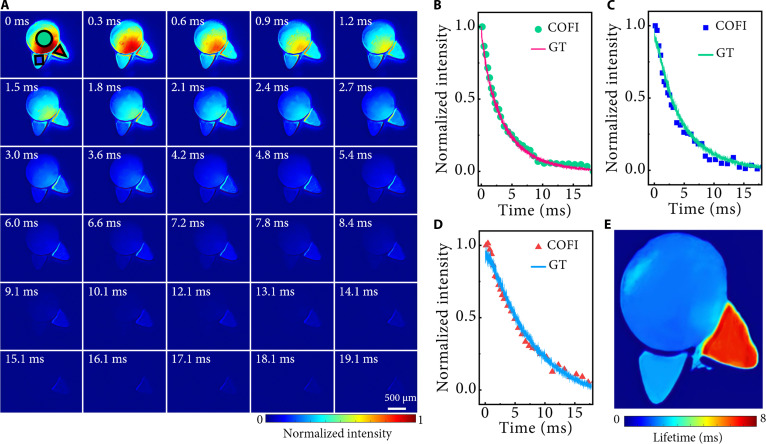
Phosphorescence lifetime microscopy of multicomponent nanoparticles with NIR-II COFI. (A) Reconstruction results using the DPNet-ESRGAN algorithm, showing a time lapse of phosphorescence intensity maps for a composite pattern containing materials with varying lifetimes. (B), (C), and (D) show the averaged and normalized phosphorescence lifetimes from reconstruction compared with ground truths for the marked circle, square, and triangle regions of interest (ROIs) in (A), respectively. (E) Phosphorescence lifetime distribution map reconstructed from the time-lapse intensity maps in (A).

Phosphorescence lifetime microscopy (PLIM) has become an indispensable tool for probing molecular interactions within complex microenvironments. NIR-II PLIM with few snapshots or even one snapshot can vastly improve the imaging speed of functional imaging [36]. Here, we used the NIR-II COFI system to perform PLIM of multicomponent rare-earth-doped nanoparticles in 2 consecutive exposures. Three types of nanoparticles, all emitting at a center wavelength of 1,525 nm but with different phosphorescence lifetimes, were designed as the 2 ears and the head of a cat-head mold. A 980-nm continuous-wave laser was modulated by a 50-Hz square wave with a 10% duty cycle to generate pulsed light for exciting the mold. After a single excitation pulse, we obtained 2 consecutive compressed images and then recovered the dynamics from these 2 compressed images using the proposed algorithm, as shown in Fig. 3A. Considering the characteristic exponential decay profile of nanoparticle fluorescence, featuring a rapid initial intensity drop followed by prolonged slow-decay tailing, a flexible temporal encoding strategy was adopted for efficient sampling. The first compressed image, acquired at a compression ratio of 20, was configured to densely sample the rapid decay phase, with encoding intervals of 300 μs for the first 10 codes and 600 μs for the last 10, each with a 150-μs exposure duration. The second compressed image, obtained with a compression ratio of 10, targeted the slower decay tail using 1-ms intercode intervals and 500-μs encoding durations.

To assess the accuracy of reconstruction, we compared the reconstructed phosphorescence lifetimes at 3 locations—where it was known that only one type of nanoparticle was present—with the actual fluorescence decay curves measured using the standard time-correlated single photon counting (TCSPC) method. The results are shown in Fig. 3B, C, and D, in which averaged and normalized phosphorescence lifetimes from reconstruction are compared with ground truths for the marked red, green, and blue regions of interest in Fig. 3A, respectively. The calculated phosphorescence lifetimes of the 3 nanoparticles are 3.2, 4.5, and 7.2 ms, respectively, which are consistent with the results measured by the TCSPC method. In addition, a 2-dimensional map of the mold’s phosphorescence lifetime was also generated, as shown in Fig. 3E. The discrepancies in lifetime in certain regions of the figure are due to inadvertent cross talk among the samples during the mold fabrication process (see Note S3 for details).

### Dynamic tracking of the flow of fluorescent microspheres within microfluidic channels

**Fig. 4.**
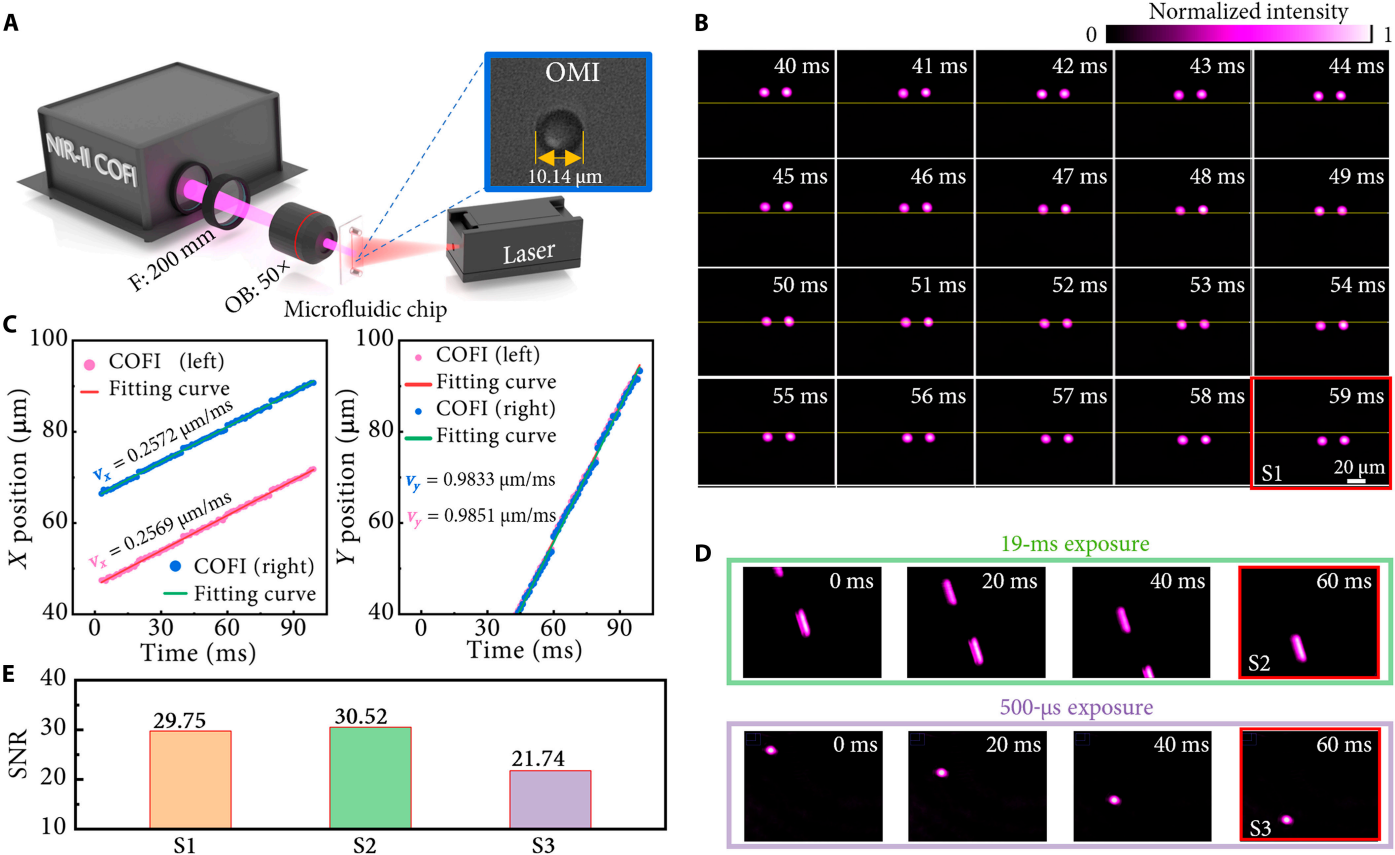
High-speed microfluidic tracking and signal-to-noise ratio (SNR) enhancement. (A) Setup for observing fluorescent microspheres in a microfluidic chip. Inset: Morphology of a single microsphere. F, lens; OB, objective lens. (B) Temporal evolution of microsphere flow (1-ms interval) reconstructed from a single snapshot. (C) The centroid displacements of the left and right microspheres in (B). (D) The results of direct imaging with an NIR-II camera under exposure times of 19 ms (top) and 500 μs (bottom). (E) The SNRs of the images within the red boxes in (B) and (D).

To further demonstrate the superiority of the NIR-II COFI system over conventional NIR-II imaging in capturing high-speed motion trajectories, we performed comparative experiments with these 2 methods. Fluorescent microspheres were suspended in deionized water and injected into a custom straight-channel microfluidic chip (50-μm width, 20-μm depth) using a syringe pump (MesoBioSystem, MS101P) at a flow rate of 0.06 μl/min. See details of the characterization of infrared fluorescent microspheres in Note S4. The morphology of a microsphere was characterized using a commercial optical microscope in advance, and the diameter was determined to be 10.14 μm, as shown in the inset of Fig. 4A. To observe the dynamic motion of fluorescent microspheres, a 50× objective lens was introduced into the system for magnification. The microfluidic channel was subsequently illuminated with continuous-wave 980-nm excitation light to induce NIR-II fluorescence emission from the microspheres, which was subsequently captured by the NIR-II COFI system. The system acquired sequential compressed measurements at a compression ratio of 20 with a coding interval of 1 ms, enabling high-speed video reconstruction at an effective frame rate of 1,000 fps. A representative set of 20 consecutive reconstructed frames is presented in Fig. 4B (see Movie S1 for the complete reconstruction). Based on all reconstructions, we calculated the temporal trajectory of the microsphere’s centroid and fitted its variation curve, as shown in Fig. 4C. The flow velocity of the particles derived from the fitted trajectory was 1.02 μm/ms, which closely matches the preset flow rate. In addition, to compare with traditional infrared imaging, we also captured the results of direct imaging without compression under the condition of the same camera frame rate and flow rate. The upper half of Fig. 4D shows the results captured by the camera with a 19-ms exposure time, while the lower half shows the results captured under the same exposure time of 500 μs as the reconstructed frames. Compared with the results of the NIR-II COFI system, it can be seen that direct imaging has severe stretching artifacts under a 19-ms long exposure, while a 500-μs short exposure captures only sparse highlights and fails to stitch the complete trajectory. Additionally, the SNR of each image acquisition method was quantitatively compared by calculating the ratio of the mean intensity in the signal region to that in the background region. As shown in Fig. 4E, the system can achieve an SNR of 29.75 at a frame rate of 1,000 fps, which is close to that with an exposure time of 19 ms and 36% higher than that achieved by simply shortening the exposure time to 500 μs. The SNR improvement is theoretically underpinned by the multiplexing advantage in detector-noise-limited systems. NIR-II COFI integrates the dynamic signal into a single snapshot to incur the sensor’s high readout noise only once, avoiding the cumulative noise penalty inherent to frame-by-frame direct imaging. The above results comprehensively demonstrate that the system can simultaneously achieve a high temporal resolution, a high SNR, and complete trajectory acquisition under the same camera frame rate and flow rate, which is particularly critical for high-speed experiments in vivo.

### In vivo imaging of intestinal peristalsis in a mouse

**Fig. 5.**
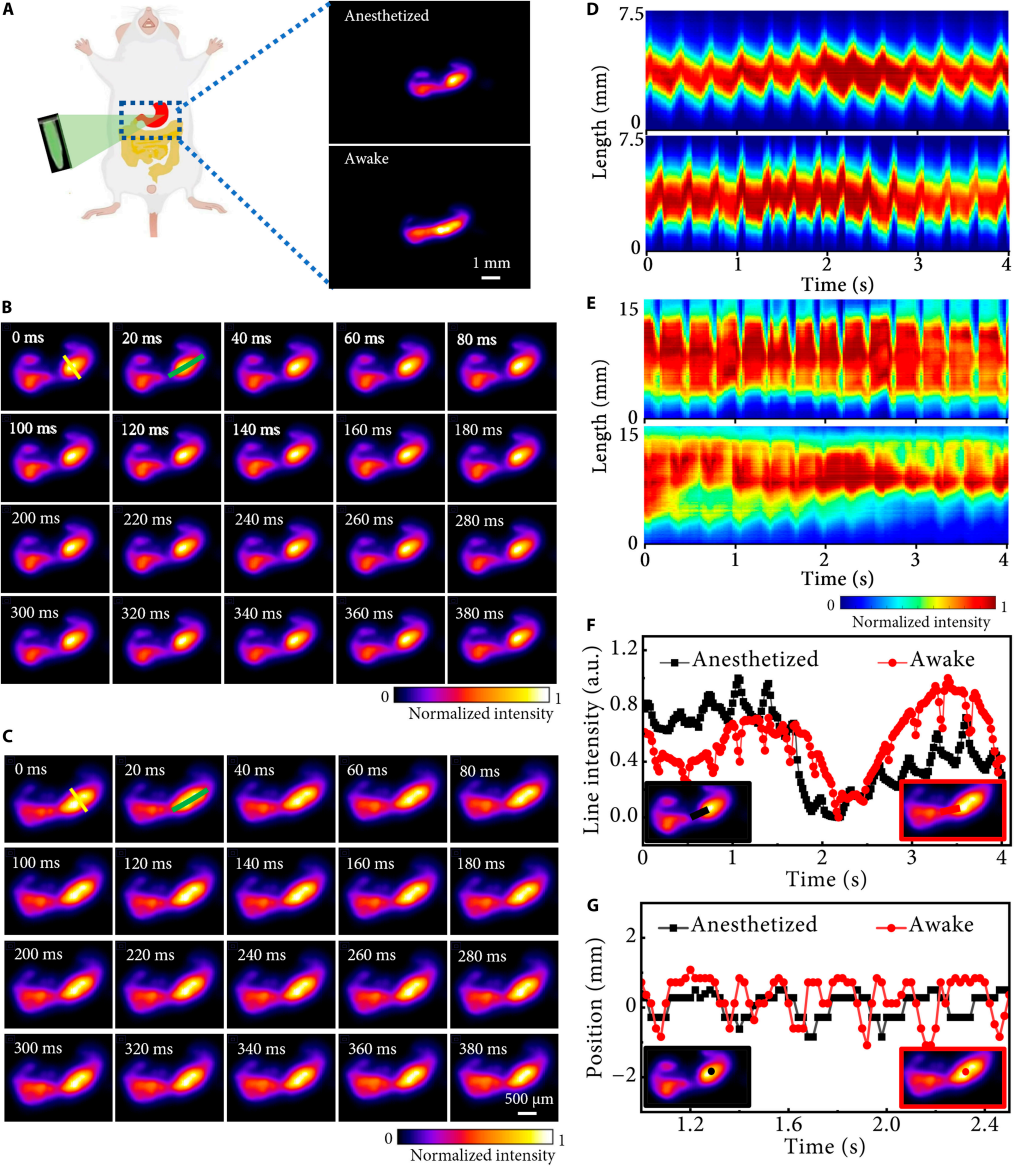
In vivo imaging of intestinal peristalsis in a mouse. (A) Luminescence images of the intestine in anesthetized and awake states, respectively. Representative reconstructed frames (B) and (C) showing the probe intensity distribution of the mouse intestine under anesthetized and awake states, respectively. (D) Spatial–temporal maps of the regions of interest (ROIs) in (B) and (C) (yellow lines). (E) Spatial–temporal maps of the ROIs in (B) and (C) (green lines). (F and G) The differences in probe intensity changes and movement of center positions in mouse intestines under different physiological conditions, respectively.

To demonstrate the system’s utility in practical NIR-II fluorescence imaging for in vivo biological studies, we conducted experiments to explore intestinal peristaltic trajectories in anesthetized and awake states, which has been a challenge for conventional imaging techniques. A depilated mouse was orally administered 200 μl of an aqueous solution containing NaYF_4_:50% Er@NaYF_4_ nanoparticles. Thirty minutes postgavage, under an NIR-II COFI optical configuration (without temporal compression), we performed single-exposure fluorescence imaging (20-ms integration) and captured the intestinal fluorescence distribution in the anesthetized state, as well as the distribution in the awake state after 15 more minutes, as shown in Fig. 5A. Subsequently, temporal compressive imaging was performed using the NIR-II COFI system to reconstruct time-resolved fluorescence distributions of intestinal motility in anesthetized and awake states; Fig. 5B and C show the reconstruction results of 2 consecutive compressed frames, respectively (see Movies S2 and S3 for the complete reconstruction results). For clarity, the reconstructed trajectories were spatially magnified (displaying 50% of the full FOV). Comparison with Fig. 5A confirms the reliability of the reconstructions. Figure 5D and E show the spatiotemporal distribution of fluorescence intensities along the midline and vertical line of the intestine in the mouse under different physiological conditions (top: anesthetized; bottom: awake), respectively. From the experimental results, it can be concluded that the intestinal movement at this stage is a wavelike motion that pushes the nanoparticles forward. Moreover, the intestinal peristalsis under anesthesia is relatively stable and slow in intensity, indicating that the movement rhythm is weak. In the awake state, the intensity fluctuations are more pronounced, indicating that the intestinal peristalsis is more active and rhythmic. This is consistent with the experimental results of Dmitriev et al. [37], which confirms the reliability of our experimental results.

Figure 5F and G depict temporal variations in fluorescence intensity and position at marked positions in their insets, revealing distinct motility patterns: respiratory and spontaneous peristaltic movements in the awake state induced periodic fluctuations in fluorescence intensity, while the anesthetized state exhibited dampened dynamics. Statistical analysis demonstrated the markedly higher frequency and amplitude of intestinal motility in the awake state versus those of the anesthetized state, consistent with prior findings reported [38]. These results validate the system’s capability for high-fidelity in vivo NIR-II imaging, providing a powerful tool for quantifying dynamic physiological processes in biomedical research.

## Discussion

By leveraging time-domain encoding to capture dynamic scenes as a compressed snapshot in a single long exposure, the developed NIR-II COFI system exhibits marked advantages in photon-budget-efficient imaging, demonstrating its transformative potential for advancing NIR-II fluorescence imaging for high-temporal-resolution in vivo observation applications. Experimental results validate that the system attains a frame rate of 3.3 kfps, representing a 20-fold improvement over conventional NIR-II frame rates, while maintaining a high SBTP of up to 4.22 × 10^8^ pixels/s. The system’s capabilities were further substantiated through diverse applications, including successfully achieving NIR-II phosphorescence lifetime imaging of multicomponent samples and clearly distinguishing lifetime differences across spatial locations. In comparative experiments involving fluorescent microsphere flow, the system demonstrated an enhanced capability to capture the trajectories of fast-moving objects, achieving a 36% SNR improvement compared to direct short-exposure imaging. Furthermore, the system successfully visualized the dynamic process of intestinal peristalsis in mice under both anesthetized and awake conditions, highlighting its substantial value for biomedical research.

Nevertheless, there remains substantial room for system optimization. The current SLM-based implementation introduces a 40% reduction in optical throughput (limiting transmission efficiency to 60%), which slightly constrains photon collection efficiency. To address this limitation, future studies could adopt motor-controlled transmissive coding architectures, such as rotating transmissive masks, for temporal compressive imaging. Such designs are projected to enhance optical throughput to >85% while preserving spatiotemporal encoding flexibility, which is particularly advantageous for deep-tissue NIR-II imaging requiring a high signal-to-background ratio. Additionally, the computational latency of the current image reconstruction algorithm precludes real-time visualization. To mitigate this, advanced deep learning architectures can be integrated to simplify the computational workflow, such as lightweight transformer networks or neural architecture search. These optimizations aim to reduce processing time to subsecond levels while maintaining reconstruction fidelity, thereby enabling real-time monitoring of dynamic physiological processes.

## Methods

### Data acquisition implementation of NIR-II COFI

**Fig. 6.**
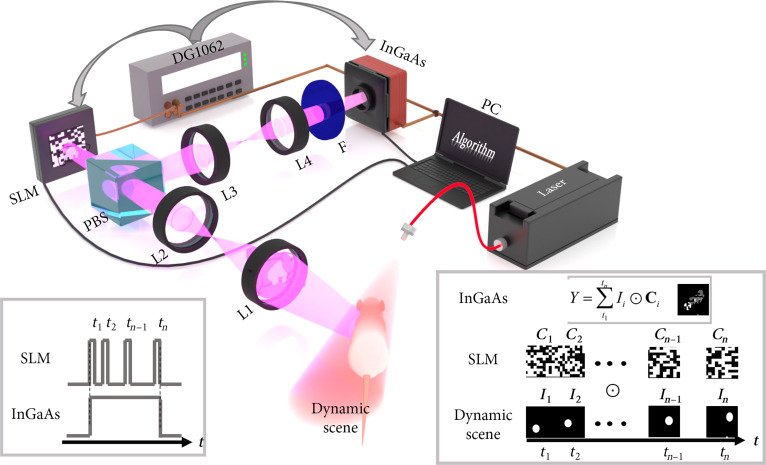
Experimental arrangement of NIR-II COFI. L, lens; Laser, 980-nm continuous-wave laser; PBS, polarizing beam splitter; SLM, spatial light modulator; DG1062, signal generator (used for synchronizing the SLM and NIR-II camera); InGaAs, NIR-II camera; F, filter, 1,400-nm long pass.

The experimental configuration of the NIR-II COFI system is shown in Fig. 6. The dynamic scene is relayed through a 4*f* optical system composed of infrared doublet lenses with focal lengths of 250 and 100 mm. A polarizing beam splitter (PBS; Lbtek PBS654) filters the incoming light from the dynamic scene into horizontally polarized components, which are subsequently imaged onto a liquid crystal-based programmable SLM (Forth Dimension Displays, QXGA R-11). Preset spatially random coding patterns are loaded onto the SLM at an ultrahigh switching rate, enabling efficient spatiotemporal encoding of the dynamic scene. The encoded dynamic scene is subsequently focused onto the imaging plane through an imaging system with focal lengths of 75 and 100 mm and finally captured by an NIR-II camera. The camera acquires multiple compressed-measurement frames for subsequent reconstruction.

The core optical modulation is jointly implemented by the SLM and the PBS. The PBS filters incident light from the dynamic scene into horizontally polarized light, while the SLM modulates polarization state variations by changing the arrangement of liquid crystal molecules to form a spatially undersampled matrix. When the modulated polarized light passes through the PBS again (which transmits light of only specific polarization orientation), the resulting intensity distribution corresponds to the coding patterns on the SLM. Notably, the high-speed programmable SLM employed offers a switching speed of 3.3 kHz, with programmable control over pattern duration to achieve adjustable sampling intervals for dynamic scenes.

### Image reconstruction of NIR-II COFI

The reconstruction framework is uniformly applied to all compressed measurements. For clarity, the theoretical description is presented using single-frame reconstruction as a representative example. The NIR-II COFI system consists of 2 main components: data acquisition and image reconstruction. The data acquisition process of the NIR-II COFI system, as shown in Fig. 6, can be mathematically formulated using operators. First, the 3D dynamic scene $I\left(x,\;y,\;t\right)$ is spatiotemporally encoded by the SLM. Specifically, the SLM sequentially loads distinct coding patterns $C_{i}\left(x,\;y,\;t_{i}\right)$ at different time points $t_{i}$ through program-controlled switching, thereby the dynamic scene is modulated over time by temporally varying spatial encodings, $I_{i}^{\prime }\left(x,\;y,\;t_{i}\right)=I_{i}\left(x,\;y,\;t_{i}\right)⊙C_{i}\left(x,\;y,\;t_{i}\right)$, in which ⊙ denotes element-wise product. Finally, the captured image represents the spatiotemporal integration of each pixel over the corresponding exposure duration. The resulting compressed-measurement data can be expressed as [39]

$$\begin{array}{c} Y\left(x,\;y\right)=\\ \sum_{t_{1}}^{t_{n}}I_{i}^{\prime }\left(x,\;y,\;t_{i}\right)=\sum_{t_{1}}^{t_{n}}I_{i}\left(x,\;y,\;t_{i}\right)⊙C_{i}\left(x,\;y,\;t_{i}\right)=\Phi I\left(x,\;y,\;t\right), \end{array}$$ (1)

where Φ is the sensing matrix. For convenience, $I\left(x,\;y,\;t\right)$ and $Y\left(x,\;y\right)$ are abbreviated as ***x*** and ***y***, respectively. Thus, the image acquisition process of NIR-II COFI can be expressed as follows:

y=Φx+n. (2)

Here, ***n*** is the noise distribution.

In the image reconstruction stage, the original dynamic scene is recovered from the observed 2-dimensional image Y and the set of encoding patterns **C** loaded for the dynamic scene by solving the inverse problem of Eq. (2). However, the inverse problem is ill posed. Traditional compressive-sensing-based optimization algorithms typically introduce a regularization term $R\left(x\right)$ to constrain the solution within a plausible signal space. The optimal estimation $\hat{x}$ of x can be found by minimizing the objective function $f\left(x\right)$, and its expression is as follows [40]:

$$\begin{array}{c} \hat{x}=\arg\min_{x}f\left(x\right)=\arg\min_{x}\left\{\frac{1}{2}{\left\|y\;-\;\Phi x\right\|}_{2}^{2}\;+\;\lambda R\left(x\right)\right\}, \end{array}$$ (3)

where **||**•**||**_2_ is the *l*_2_ norm, ${\left\|y-\Phi x\right\|}_{2}^{2}$ represents the fidelity term that the reconstructed scene needs to conform to the sampling equation, $R\left(x\right)$ represents the regularization term that the reconstructed scene needs to satisfy the prior information, and λ is the regularization parameter that balances these 2 terms. Here, the problem is solved based on a PnP-ADMM framework [41]. In this framework, by introducing the auxiliary variable ***θ***, Eq. (3) can be rewritten as

$$\min_{\theta \;,x}\frac{1}{2}{\left\|y\;-\;\Phi x\right\|}_{2}^{2}+\lambda R\left(\theta \right)\;s.t.\;\theta =x.$$ (4)

According to the augmented Lagrangian (AL) multiplier method, the optimal solution of Eq. (4) can be obtained by minimizing an AL function, which is expressed as

$$L_{\rho }\left(x,\;\theta ,\;u\right)=\frac{1}{2}{\left\|y\;-\;\Phi x\right\|}_{2}^{2}+\lambda R\left(\theta \right)+u^{T}\left(\theta \;-\;x\right)\;-\frac{\rho }{2}{\left\|\theta \;-\;x\right\|}_{2}^{2},$$ (5)

where ***u*** is another auxiliary variable and ρ is the penalty parameter. By using the ADMM framework, the minimization process of $L_{\rho }$ can be decomposed into the following 3 subproblems [42]:

$$x_{k+1}=\underset{x}{\arg\min}\frac{1}{2}{\left\|y\;-\;\Phi x\right\|}_{2}^{2}+\frac{\rho }{2}{\left\|x-\left(\theta_{k}-\frac{1}{\rho }u_{k}\right)\right\|}_{2}^{2},$$ (6)

$$\theta_{k+1}=\;\underset{\theta }{\arg\min}\lambda R\left(\theta \right)+\frac{\rho }{2}{\left\|\theta \;-\left(x_{k+1}+\frac{1}{\rho }u_{k}\right)\right\|}_{2}^{2},$$ (7)

$$u_{k+1}=u_{k}+\rho \left(x_{k+1}-\theta_{k+1}\right).$$ (8)

Here, the superscript *k* represents the *k*th iteration. Equation (6) is a quadratic equation, and its closed-form solution is [43]

$$x_{k+1}=D_{\sigma }{\left[\Phi^{T}\Phi +\rho x\right]}^{-1}\left[\Phi^{T}y+\theta_{k}-\frac{1}{\rho }u_{k}\right]=G\;\left(•\right),$$ (9)

where **D** is the denoising algorithm used and *σ* is the standard deviation of the noise [38]

$$\theta_{k+1}=D_{\sigma }\left[x_{k+1}+\frac{1}{\rho }u_{k}\right].$$ (10)

**Fig. 7.**
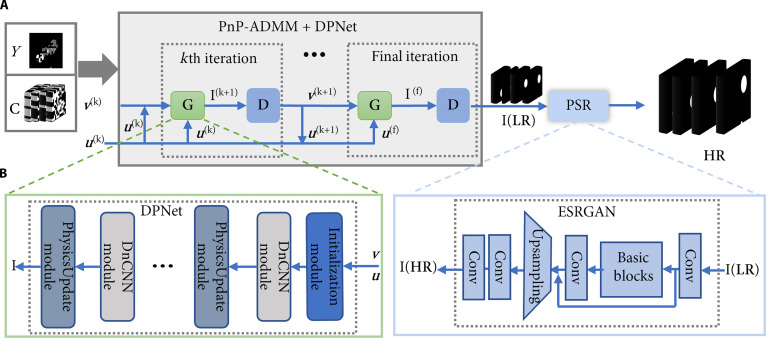
The image reconstruction and pixel super-resolution flowchart of NIR-II COFI. (A) Image reconstruction diagram of the DPNet-ESRGAN algorithm. (B) Solver G(·) in a sparse domain (left) and the network of the ESRGAN module (right). PnP-ADMM, plug-and-play alternating direction method of multipliers; LR, low-resolution image; PSR, pixel super-resolution; HR, high-resolution image; Conv, convolutional layer.

The schematic of the proposed DPNet-ESRGAN algorithm is illustrated in Fig. 7A. Initially, the encoded patterns **C** and the observed image Y are input into the PnP-ADMM-DPNet iterative framework, where operators the **G**(**·**) and **D** represent the operations of Eqs. (9) and (10), respectively. To enhance denoising performance, a cascaded denoising configuration, with total variation, fast and flexible denoising network (FFDNet) [44], denoising residual U-net (DRUNet) [45], and fast deep video denoising network (FastDVDNet) [46] in sequence, is employed. FFDNet and DRUNet utilize a noise level map as auxiliary input to effectively denoise individual reconstructed frames, while FastDVDNet exploits temporal neighborhood information to improve the temporal consistency of residual noise, thereby substantially enhancing overall reconstruction quality. After a finite number of iterations, the reconstructed ***Y*** enters the PSR module based on an ESRGAN [47], yielding high-fidelity spatiotemporal evolution of the original dynamic scene.

To further improve reconstruction accuracy, DPNet [48] (see details in Note S6) that outperforms traditional algorithms is utilized to obtain **G**(**·**). The main body of this network is composed of the Initialization module, and alternant DnCNN [48] and PhysicsUpdate modules, as shown in Fig. 7B (left). The processed dataset is fed into the DPNet network to learn the encoding behavior of NIR-II COFI, thereby obtaining the solver **G**(**·**). Compared to traditional algorithms like the 2-step iterative shrinkage/thresholding algorithm, AL, and PnP-ADMM, our algorithm optimizes the sparse domain and related iterative parameters $\left\{\rho ,\;\theta_{k},\;u_{k}\right\}$ through end-to-end training. Being inherently data driven, the framework leverages the powerful nonlinear representation capacity of deep neural networks to learn complex mappings from large-scale datasets. By optimizing the sparse domain during training, the algorithm effectively reduces both sparsity and mutual coherence, enabling more accurate and high-fidelity reconstructions.

Furthermore, to overcome the inherent spatial resolution limitations of NIR-II imaging caused by its longer wavelength and compensate for potential information loss during the compressive-sensing-based reconstruction in NIR-II COFI, a pixel-level super-resolution module based on the ESRGAN is also utilized. As depicted in Fig. 7B (right), the network begins with an initial convolutional layer for basic feature extraction and then processes them through the residual-in-residual dense blocks (RRDBs), which combine dense connections and nested residuals to enhance feature learning and mitigate gradient vanishing. These RRDBs effectively capture multiscale contextual features, enabling the network to extract rich texture details from the input image. Spatial resolution is improved through either subpixel convolution or transposed convolution operations in the upsampling module, ensuring the precise reconstruction of high-frequency components. Post-upsampling refinement layers further optimize the features to eliminate artifacts and improve structural coherence, ultimately producing high-resolution outputs. The framework employs a combination of hybrid loss functions, including perceptual loss, adversarial loss, and content loss, to generate super-resolved images with photorealistic textures, minimized distortion, and enhanced fidelity, addressing the resolution degradation challenges inherent in NIR-II COFI.

## Data Availability

All relevant data are available from the authors on request.

## Supplementary Materials

Supplementary 1 Notes S1 to S6 Table S1 Figs. S1 to S8 Movies S1 to S3

## References

[1] Jöbsis FF. Noninvasive, infrared monitoring of cerebral and myocardial oxygen sufficiency and circulatory parameters. Science. 1977;198(4323):1264–1267. 929199 10.1126/science.929199

[2] Hong G, Wu JZ, Robinson JT, Wang H, Zhang B, Dai H. Three-dimensional imaging of single nanotube molecule endocytosis on plasmonic substrates. Nat Commun. 2012;3(1):700. 22426221 10.1038/ncomms1698

[3] Hong G, Lee JC, Robinson JT, Raaz U, Xie L, Huang NF, Cooke JP, Dai H. Multifunctional *in vivo* vascular imaging using near-infrared II fluorescence. Nat Med. 2012;18(12):1841–1846. 23160236 10.1038/nm.2995 PMC3595196

[4] Sadeghi SA, Fang F, Tabatabaeian Nimavard R, Wang Q, Zhu G, Saei AA, Sun L, Mahmoudi M. Mass spectrometry-based top-down proteomics for proteoform profiling of protein coronas. Nat Protoc. 2025;20:2560–2585. 40764671 10.1038/s41596-025-01229-6 PMC12407567

[5] Yu X, Zeng L, Yang X, Ren Z, Dong X, Meng G, Shan G, Yan D, Wang D, Sun F. An NIR-II absorbing injectable hydrogel for boosted photo-immunotherapy. Aggregate. 2025;6(4): Article e743.

[6] Lin D, Huang W, Yang H, Zhu J, Liu Y, Wang L, Yan D, Wang D, Tang BZ. Ultrabright NIR-II nanoparticles for high-resolution in vivo imaging. Adv Mater. 2025;37: Article e10493. 10.1002/adma.202510493 41165516

[7] Chang B, Li D, Ren Y, Qu C, Shi X, Liu R, Liu H, Tian J, Hu Z, Sun T, et al. A phosphorescent probe for in vivo imaging in the second near-infrared window. Nat Biomed Eng. 2022;6(5):629–639. 34385694 10.1038/s41551-021-00773-2

[8] Roy S, Bag N, Bardhan S, Hasan I, Guo B. Recent progress in NIR-II fluorescence imaging-guided drug delivery for cancer theranostics. Adv Drug Deliv Rev. 2023;197: Article 114821. 37037263 10.1016/j.addr.2023.114821

[9] Cao J, Zhu B, Zheng K, He S, Meng L, Song J, Yang H. Recent progress in NIR-II contrast agents for biological imaging. Front Bioeng Biotechnol. 2020;7:487. 32083067 10.3389/fbioe.2019.00487 PMC7002322

[10] Miao Q, Pu K. Organic semiconducting agents for deep-tissue molecular imaging. Adv Mater. 2018;30(49):1801778. 10.1002/adma.201801778 30058244

[11] Bosschaart N, Edelman GJ, Aalders MC, van Leeuwen T, Faber DJ. Optical properties of whole blood: A literature review and theoretical approach. Lasers Med Sci. 2014;29(2):453–479. 24122065 10.1007/s10103-013-1446-7 PMC3953607

[12] Kou L, Labrie D, Chylek P. Refractive indices of water and ice in the 0.65–2.5 μm spectral range. Appl Opt. 1993;32(19):3531–3540. 20829977 10.1364/AO.32.003531

[13] Bashkatov AN, Genina EA, Kochubey VI, Tuchin VV. Optical properties of subcutaneous adipose tissue in the 400–2500 nm spectral range. Opt Spectrosc. 2005;99(5):836–842.

[14] Diao S, Hong G, Antaris AL, Blackburn JL, Cheng K, Cheng Z, Dai H. Biological imaging without autofluorescence in the second near-infrared region. Nano Res. 2015;9:3027–3034.

[15] Hong G, Antaris AL, Dai H. Near-infrared fluorophores for biomedical imaging. Nat Biomed Eng. 2017;1(1):0010.

[16] Zhang X, Li S, Ma H, Wang H, Zhang R, Zhang XD. Activatable NIR-II organic fluorescent probes for bioimaging. Theranostics. 2022;12(7):3345–3362. 35547762 10.7150/thno.71359 PMC9065193

[17] Ullah Z, Roy S, Hasan I, Madni M, Gong T, Roy J, Sau A, Yan Y, Soe SK, Zhang Y, et al. NIR-II fluorescence and MRI-guided piezodynamic therapy using a biomimetic nanoplatform. Small. 2025;21(48): Article e10697. 41070788 10.1002/smll.202510697

[18] Yu X, Feng Z, Cai Z, Jiang M, Xue D, Zhu L, Zhang Y, Liu J, Que B, Yang W, et al. Deciphering cerebrovasculature via ICG-assisted NIR-II fluorescence microscopy. J Mater Chem B. 2019;7(42):6623–6629. 31591622 10.1039/c9tb01381d

[19] Li S, Deng X, Cheng H, Li X, Wan Y, Cao C, Yu J, Liu Y, Yuan Y, Wang K, et al. Bright near-infrared π-conjugated oligomer nanoparticles for deep-brain three-photon microscopy. ACS Nano. 2022;16(8):12480–12487. 35968934 10.1021/acsnano.2c03813

[20] Schmidt EL, Ou Z, Ximendes E, Cui H, Keck CH, Jaque D, Hong G. Near-infrared II fluorescence imaging. Nat Rev Methods Primers. 2024;4(1):23.

[21] Welsher K, Liu Z, Sherlock SP, Robinson JT, Chen Z, Daranciang D, Dai H. Brightly fluorescent carbon nanotubes for near-infrared imaging in mice. Nat Nanotechnol. 2009;4(11):773–780. 19893526 10.1038/nnano.2009.294 PMC2834239

[22] Huang H, Li M, Gu J, Roy S, Jin J, Kuang T, Zhang Y, Hu G, Guo B. Bright NIR-II emissive cyanine dye-loaded nanoparticles for imaging-guided photothermal therapy. J Nanobiotechnol. 2024;22(1):788. 10.1186/s12951-024-03074-3 PMC11665114 39710705

[23] Liu Y, Li M, Gu J, Huang H, Xie H, Yu C, Roy S, Chen X, Kuang T, Zhang Y, et al. Exosome–liposome hybrid nanomedicines for NIR-II imaging-guided photothermal therapy. Colloids Surf B Biointerfaces. 2025;245: Article 114258. 39303384 10.1016/j.colsurfb.2024.114258

[24] Yu C, Hu Z, Hu G, Jia Q, Xiao Y, Ahmad H, Zhang D, Liu Z, Iqbal MS, Zeng W, et al. NIR-II fluorescence membrane probes for targeted photothermal–immunotherapy. Biomaterials. 2025;305: Article 123770. 10.1016/j.biomaterials.2025.123770 41092647

[25] Dong B, Li C, Chen G, Zhang Y, Zhang Y, Deng M, Wang Q. Highly photoluminescent Ag_2_Se quantum dots for NIR-II in vivo imaging. Chem Mater. 2013;25(12):2503–2509.

[26] Fan Y, Wang P, Lu Y, Wang R, Zhou L, Zheng X, Li X, Piper JA, Zhang F. Lifetime-engineered NIR-II nanoparticles unlock multiplexed in vivo imaging. Nat Nanotechnol. 2018;13(10):941–946. 30082923 10.1038/s41565-018-0221-0

[27] Biswas B, More P, Kandula HN. Emergent softening and stiffening dictate transport of active colloidal filaments. ACS Nano. 2025;19(34):31038–31049. 40828683 10.1021/acsnano.5c08920

[28] Greiner J, Sankarankutty AC, Seidel T, Sachse FB. Confocal microscopy-based estimation of intracellular conductivities. Comput Biol Med. 2022;146: Article 105579. 35588677 10.1016/j.compbiomed.2022.105579 PMC10195095

[29] Wang F, Wan H, Ma Z, Zhong Y, Sun Q, Tian Y, Qu L, du H, Zhang M, Li L, et al. Light-sheet microscopy in the near-infrared II window. Nat Methods. 2019;16(6):545–552. 31086342 10.1038/s41592-019-0398-7 PMC6579541

[30] Wang F, Ma Z, Zhong Y, Salazar F, Xu C, Ren F, Qu L, Wu AM, Dai H. In vivo NIR-II structured-illumination light-sheet microscopy. Proc Natl Acad Sci USA. 2021;118(6): Article e2023888118. 33526701 10.1073/pnas.2023888118 PMC8017937

[31] Zubkovs V, Antonucci A, Schuergers N, Lambert B, Latini A, Ceccarelli R, Santinelli A, Rogov A, Ciepielewski D, Boghossian AA. Spinning-disc confocal microscopy in the second near-infrared window. Sci Rep. 2018;8(1):13770. 30214049 10.1038/s41598-018-31928-y PMC6137042

[32] Zhang H, Zhu L, Gao DS, Liu Y, Zhang J, Yan M, Qian J, Xi W. Imaging deep spinal cord microvasculature with high-speed NIR-II microscopy. Small Methods. 2022;6(8):2200155. 10.1002/smtd.202200155 35599368

[33] Li B, Niu Y, Feng Y. Thermal radiation effects in near-infrared single-photon detectors. Optoelectron Lett. 2023;19(8):468–471.

[34] Teledyne Princeton Instruments. NIRvana 640 SWIR camera. Teledyne Princeton Instruments. 2026. [accessed 22 Feb 2026] https://www.teledynevisionsolutions.com/products/nirvana/?model=NRV-640&vertical=tvs-princeton-instruments&segment=tvs

[35] Tang J, Davies MA. A fast stochastic plug-and-play ADMM for imaging inverse problems. arXiv. 2020. 10.48550/arXiv.2006.11630

[36] Zhu X, Wang X, Zhang H, Zhang F. Luminescence lifetime imaging based on lanthanide nanoparticles. Angew Chem Int Ed Engl. 2022;61(42): Article e202209378. 35918764 10.1002/anie.202209378

[37] Dmitriev RI, Intes X, Barroso MM. Luminescence lifetime imaging of three-dimensional biological objects. J Cell Sci. 2021;134(9):jcs258569. 10.1242/jcs.254763 PMC8126452 33961054

[38] Chen ZH, Yun B, Hou Y, Wang X, Wang X, Xu J, Jiang L, Han T, Zhang H, Zhang F. NIR-II anti-Stokes luminescence nanocrystals with 1710 nm excitation for in vivo bioimaging. Angew Chem Int Ed Engl. 2025;64(4): Article e202416893. 39382037 10.1002/anie.202416893

[39] Yang C, Cao F, Qi D, He Y, Ding P, Yao J, Jia T, Sun Z, Zhang S. Hyperspectrally compressed ultrafast photography. Phys Rev Lett. 2020;124(2): Article 023902. 32004022 10.1103/PhysRevLett.124.023902

[40] Figueiredo MA, Nowak RD, Wright SJ. Gradient projection for sparse reconstruction: Application to compressed sensing and other inverse problems. IEEE J Sel Top Signal Process. 2007;1(4):586–597.

[41] Chan SH, Wang X, Elgendy OA. Plug-and-play ADMM for image restoration. IEEE Trans Comput Imaging. 2016;3(1):84–98.

[42] Boyd S, Parikh N, Chu E, Peleato B, Eckstein J. Distributed optimization and statistical learning via the alternating direction method of multipliers. Found Trends Mach Learn. 2011;3(1):1–122.

[43] Liu Y, Yuan X, Suo J, Brady DJ, Dai Q. Rank minimization for snapshot compressive imaging. IEEE Trans Pattern Anal Mach Intell. 2019;41(12):2990–3006. 30295611 10.1109/TPAMI.2018.2873587

[44] Zhang K, Zuo W, Zhang L. FFDNet: Toward a fast and flexible solution for CNN-based image denoising. IEEE Trans Image Process. 2018;27(9):4608–4622. 10.1109/TIP.2018.2839891 29993717

[45] Zhang K, Li Y, Zuo W, Zhang L, van Gool L, Timofte R. Plug-and-play image restoration with deep denoiser prior. IEEE Trans Pattern Anal Mach Intell. 2022;44(10):6360–6376. 34125670 10.1109/TPAMI.2021.3088914

[46] Tassano M, Delon J, Veit T. DVDnet: A fast network for deep video denoising. Paper presented at: 2019 IEEE International Conference on Image Processing (ICIP); 2019 Sep 22–25; Taipei, Taiwan.

[47] Wang X, Xie L, Dong C, Shan Y. Real-ESRGAN: Training real-world blind super-resolution with pure synthetic data. Paper presented at: 2021 IEEE/CVF International Conference on Computer Vision Workshops (ICCVW); 2021 Oct 11–17; Montreal, Canada.

[48] Dong W, Wang P, Yin W, Shi G, Wu F, Lu X. Denoising prior driven deep neural network for image restoration. IEEE Trans Pattern Anal Mach Intell. 2019;41(10):2305–2318. 30295612 10.1109/TPAMI.2018.2873610

