# Supplementary material for: High-Speed, Pixel-Super-resolved Compressive Second Near-Infrared Fluorescence In Vivo Imaging
Source: Research (Wash D C). 2026 Mar 19;9:1146. doi: 10.34133/research.1146 (PMC13000113; doi:10.34133/research.1146)
Supplement: Supplementary 1 — Notes S1 to S6 Table S1 Figs. S1 to S8 Movies S1 to S3 [file research.1146.f1.zip › NIR-II COFI_0116_SI anonymous DQr.docx]

***Supplementary Information for***

**High-speed, pixel-super-resolved compressive NIR-II fluorescence *in-vivo* imaging**

**Supplementary Note 1 Performance comparison of various NIR-II fluorescence imaging methods.**

**Table s1** Comparison of key performance parameters between the proposed NIR-II COFI system and existing NIR-II imaging techniques.

| Imaging Modality | Typical Frame Rate | SBTP (pixels/s) | Relative Spatial Resolution | Relative SNR | Primary Limitation |
| --- | --- | --- | --- | --- | --- |
| NIR-II MultiPhoton Microscopy[S1] | < 5 fps | <2.4 × 10⁶ | Medium | High (Point scanning) | Speed-throughput trade-off |
| NIR-II Confocal Microscopy (Spinning-disc)[S2] | <20 fps | <1.3 × 10⁷ | High | High (Point scanning) | Low temporal resolution due to mechanical scanning |
| NIR-II Light-Sheet (LSM)[S3] | < 20 fps* | <1.5 × 10⁷ | High | High (Optical sectioning) | Complex illumination; speed limited by camera readout |
| NIR-II SIM[S4] | < 10 fps | <5.0 × 10⁶ | Extremely high (exceed diffraction limit) | Medium | Very low temporal resolution (requires multi-phase exposure) |
| NIR-II Camera (e.g., NIRvana 640)[S5] | 110 fps | 3.6 × 10⁷ | Medium | Medium (at high speeds due to short exposure) | Speed-SNR trade-off; limited by readout speed |
| NIR-II COFI (This Work) | **3,300 fps** | **4.22 × 10⁸** | Medium | Medium (**+36% vs. direct**) | Computational reconstruction time |

******* *LSM is inherently a volumetric (3D) imaging modality. The frame rate listed here refers to the acquisition speed for a single 2D optical section as reported in Ref. [S3]. The effective volumetric imaging speed is significantly lower depending on the number of z-slices.*

Currently, advanced in vivo NIR-II imaging technologies primarily include confocal microscopy, light-sheet microscopy (LSM), structured illumination microscopy (SIM), and multiphoton microscopy. We have summarized the key performance parameters of these techniques and developed the NIR-II COFI system in Table s1.

Specifically, confocal microscopy, multiphoton microscopy, and LSM rely on point-by-point or plane-by-plane scanning mechanisms, which inherently limit their imaging speed. Even if high-speed scanning devices are employed, the effective imaging speed and the space-bandwidth-time product (SBTP) remain constrained by the readout speed of InGaAs detectors. SIM requires the acquisition of multiple raw images with varying phases and angles to reconstruct a single frame, resulting in a relatively low temporal resolution. In contrast, NIR-II COFI technique leverages compressive sensing to achieve single-exposure imaging, enabling it to exceed the native frame rate of the camera.

Regarding SNR, confocal microscopy, multiphoton microscopy, and LSM achieve exceptional signal-to-noise ratios (SNR) by physically rejecting out-of-focus signals. The NIR-II COFI and SIM are both based on wide-field illumination and therefore generally exhibit lower SNR compared to the aforementioned scanning-based techniques. However, it is important to note that by encoding dynamic scenes into a single long-exposure frame, NIR-II COFI achieves a significant SNR improvement of approximately 36% compared to direct high-speed wide-field imaging.

Given that absolute spatial resolution is dependent on factors such as system magnification and the scattering properties of specific biological samples, the relative resolution based on the imaging principles of each technique was listed here. In this context, the spatial resolution of NIR-II COFI is comparable to that of conventional direct imaging.

**Supplementary Note 2 Characterization of NIR-II COFI system**

To begin with, the algorithm names in the supplementary materials are composed of the individual components of each architecture to clearly explain the structure of our proposed algorithms. Specifically, PnP-ADMM-DPNet refers to the version of our method without the pixel super-resolution module, while ADMM-DPNet-ESRGAN corresponds to the full version of our proposed algorithm, DPNet-ESRGAN, as presented in the main text.


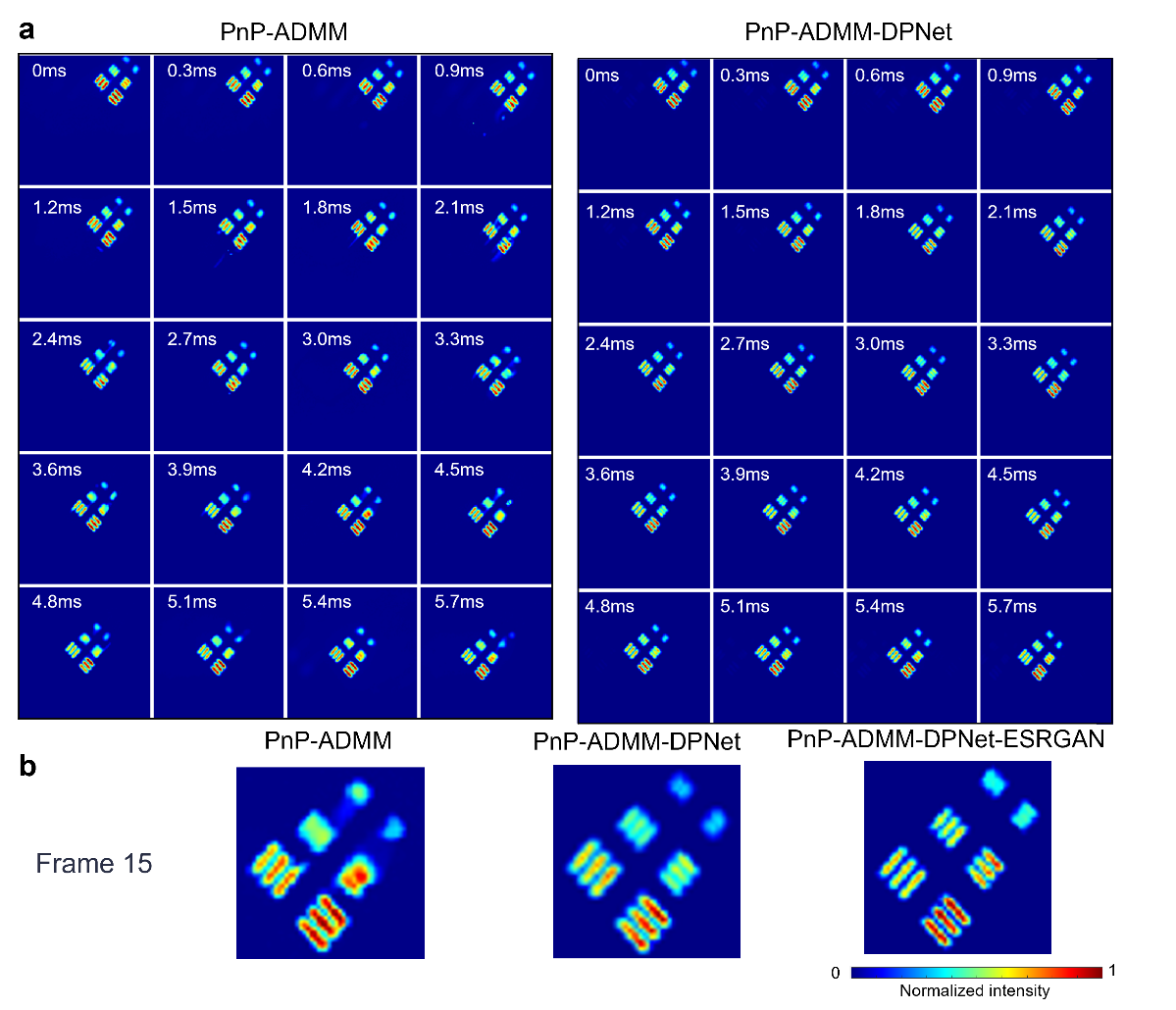


**Fig. s1** (a) The reconstruction results of the same compressed image of Fig. 2(a) using the PnP-ADMM algorithm (left) and the PnP-ADMM-DPNet algorithm (right). (b) The 15th reconstructed image from the PnP-ADMM, PnP-ADMM-DPNet, and ADMM-DPNet-ESRGAN algorithms.

To validate the superiority of the proposed algorithm, we compared the reconstruction results of the same compressed image (Fig. 2 in the main text) using the PnP-ADMM algorithm and the PnP-ADMM-DPNet, as shown in Fig. s1a. It is evident that PnP-ADMM results exhibit residual motion artifacts in several frames, whereas PnP-ADMM-DPNet yields consistently accurate and stable reconstructions. This comparison clearly demonstrates that the stability and accuracy of reconstruction are significantly improved after replacing the solver in the algorithm with the DPNet network structure. We further compared the 15th reconstructed frame produced by PnP-ADMM, PnP-ADMM-DPNet, and our proposed ADMM-DPNet-ESRGAN pipeline. Fig. s1b illustrates that the basic PnP-ADMM output shows blurred edges and low contrast in high-frequency regions. Adding DPNet reduces noise and sharpens features, but some fine details remain unresolved. Incorporating the ESRGAN super-resolution module further enhances edge delineation and contrast, leading to significant gains in image quality and spatial resolution and enabling clear visualization of the smallest line-pair features.


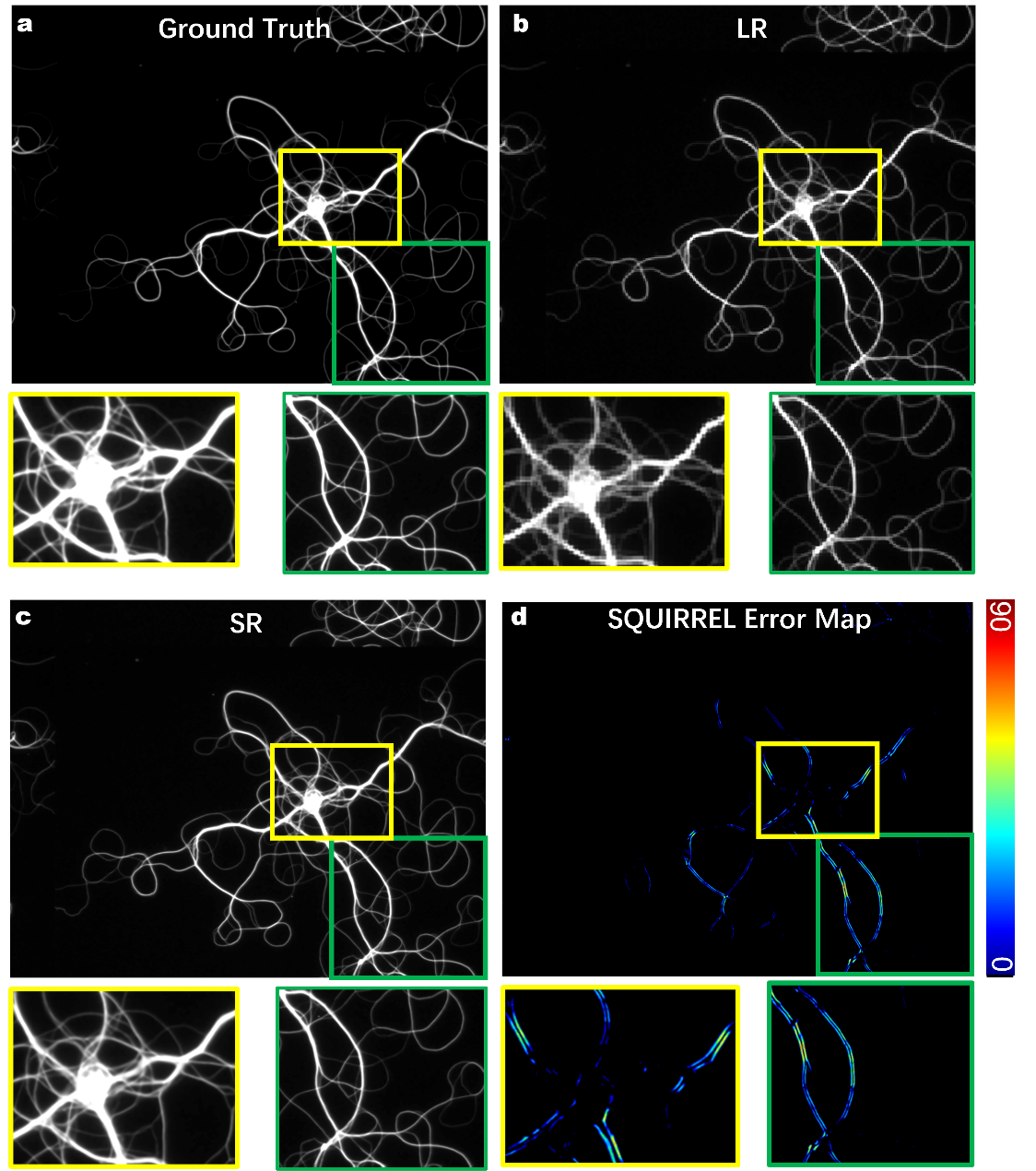


**Fig. s2** Super-resolution results produced by the ESRGAN module. (a) Original high-resolution ground truth (GT) image. (b) Degraded low-resolution (LR) input image. (c) Super-resolution (SR) image reconstructed by the ESRGAN module. (d) Error map illustrating the difference between the GT (a) and the SR result (c).

To further verify that the ESRGAN module recovers structural information from the input data rather than synthesizing it from training priors, we performed an additional blind validation test.

Here, we selected a high-resolution cell image (unseen by the network) as the ground truth, as shown in Fig. s2(a). We then mathematically simulated the degradation process by convolving this image with the optical aberrations and point spread function (PSF) inherent to our system, followed by downsampling to generate a low-quality input that mimics raw experimental capture, which is shown in Fig. s2(b). This degraded image was then fed into the ESRGAN network for blind restoration. As can be seen from Fig. s2(c), the network successfully restored fine cellular structures in the super-resolved image. A quantitative comparison between the reconstruction and the original high-resolution ground truth yielded a PSNR value of 32.49. To explicitly visualize and quantify the restoration accuracy, we employed the super-resolution quantitative image rating and reporting of error locations (SQUIRREL) method[S6], a standard approach in the super-resolution field, to generate error maps. Comparison between the error plot and the original plot in Fig. s2(d) reveals that no additional structure is produced and there is only a small difference in the intensity data at some locations. The analysis yields a resolution scaled error of 6.4937 and a resolution scaled pearson coefficient of 0.7378. These metrics confirm that the enhanced details spatially align with the actual structure without introducing non-existent artifacts.

**Supplementary Note 3 Fluorescence lifetime imaging of multi-component rare-earth-doped nanoparticles**

Fig. s3 shows the visible-light emission image of the samples under continuous-wave 980 nm laser excitation. The second-brightest red region corresponds to sample 1 (lifetime = 3.2 ms), the brightest red region corresponds to sample 2 (lifetime = 4.5 ms), and the yellow-emitting region corresponds to sample 3 (lifetime = 7.2 ms). These assignments agree with the fluorescence-lifetime distribution maps presented in the main text.


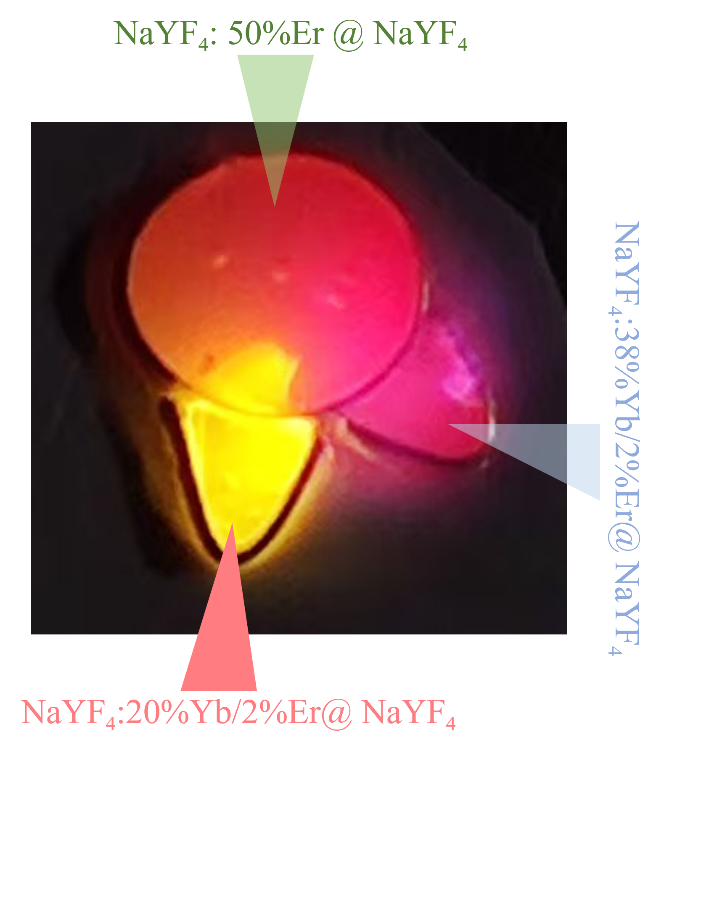


**Fig. s3** The image of the visible light emitted from the sample under the excitation of a 980 nm continuous-wave laser.

**Supplementary Note 4** **Characterization of infrared fluorescent microspheres**

In the fluorescent microbead flow experiment described in the main text, the fluorescent microbeads used were microspheres composed of NaYF₄:50%Er@NaYF₄ nanoparticles.


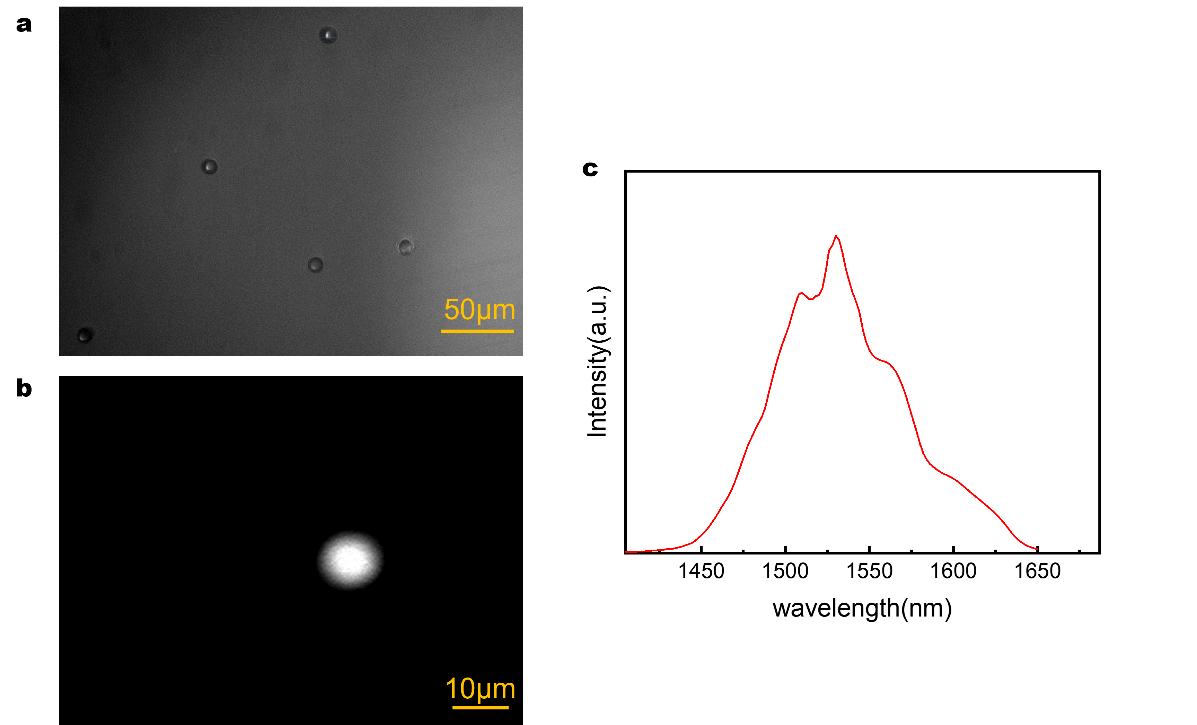


**Fig. s4** Characterization of infrared fluorescent microspheres. (a) Morphology of microspheres observed by optical microscopy. (b) Fluorescence image of the microspheres under 980 nm excitation. (c) Luminescence emission spectra of the fluorescent microspheres in the spectral range of 1400-1700 nm.

**Supplementary Note 5 Preparation and characterization of all materials used in the experiment**

**Chemicals**

Erbium (III) chloride hydrate (99.9%), ytterbium (III) chloride hydrate (99.9%), yttrium (III) chloride hydrate (99.9%), sodium trifluoroacetate (Na-TFA, 98 %), 1-octadecene (ODE, 90%), oleic acid (OA, 90%) were purchased from Sigma-Aldrich. Sodium hydroxide (NaOH, 96%), ammonium fluoride (NH4F, 96%) and ethanol were obtained from Beijing Chemical Reagents Co. Ltd. SYLGARD silicone elastomer 184 and curing agent were purchased from Dow Corning. 1,2-distearoyl-sn-glycero-3-phosphoethanolamine-N- [methoxy (polyethylene glycol)-2000] (DSPE-PEG2000-OCH3) was purchased from Shanghai Ponsure Biotech. All chemicals were used as received without any further purification.

**Preparation of shell precursors**

Y-OA (0.10 M) precursor: a mixture of YCl3 (10 mmol), OA (40.0 mL), and ODE (60.0 mL) was loaded in a reaction container and heated at 140 ℃ under vacuum with magnetic stirring for 60 min to remove residual water and oxygen. Then the colorless Y-OA precursor solution (0.10 M) was obtained.

Yb-OA (0.10 M) precursor: The synthesis of the Yb-OA precursor was similar to that of the Y-OA precursor except 10 mmol of YbCl3 were used instead of 10 mmol of YCl3.

Er-OA (0.10 M) precursor: The synthesis of the Er-OA precursor was similar to that of the Y-OA precursor except 10 mmol of ErCl3 were used instead of 10 mmol of YCl3.

Na-TFA-OA (0.40 M) precursor: A mixture of Na-TFA (40.00 mmol) and OA (100.0 mL) was loaded in a 250 mL container at room temperature under vacuum with magnetic stirring to remove residual water and oxygen. Then the colorless Na-TFA-OA precursor solution (0.40 M) was obtained.

**Synthesis of β-NaYF**4**: x% Ln (Ln = Er, Yb) core nanoparticles**

Hexagonal phase β-NaYF4 doped nanocrystals were synthesized following a previously reported method.[S7] In a typical synthesis, calculated amounts of Y(CH3CO2)3·4H2O, Ln(CH3CO2)3·4H2O to a total of 0.4 mmol, OA (3.0 mL) and ODE (7.0 mL) were mixed together and heated to 140 °C under vacuum until a clear solution formed, after that, the solution was cooled down to room temperature. To this solution at room temperature, a methanol solution (5.0 mL) of ammonium fluoride (1.6 mmol) and sodium hydroxide (1 mmol) was added and stirred for 1 h at 50 °C. The temperature was then increased to 100°C to remove methanol and water from the reaction mixture under vacuum for 30 min. Then the reaction mixture was heated to 290 °C (∼10 °C/min) and maintained for 90 min under a gentle argon flow. After the solution was cooled down to room temperature, the resulting nanoparticles were precipitated out by the addition of ethanol, and washed twice with ethanol. The nanoparticles were finally dispersed in 4 mL of cyclohexane for further use.

**β-NaYF4: x% Ln@NaYF4 core/shell nanoparticles**

In a typical process, 3 mL of the purified targeted core cyclohexane solution (0.1M) was mixed with 4.8 mL OA and 7.2 mL ODE. The flask was pumped down at 100 °C for 30 min to remove cyclohexane and any residual air and water. Subsequently, the reaction mixture was switched to Ar flow, and further heated to 280 °C at a rate of ~ 20 °C/min. Then, the well-mixed Ln (0.1 M) and Na-TFA-OA (0.4 M) host shell precursors were simultaneously introduced by dropwise addition at 280 °C. The shell thickness can be well tuned by changing the amount of the shell precursors. The amount of Ln-OA precursor was no more than 3 times that of core. Finally, the obtained core/shell nanoparticles were precipitated and washed in the same way as the core nanoparticles and dispersed in cyclohexane.

**Fabrication of the luminescent lifetime samples**

The nanocrystals (1 mmol) in cyclohexane were firstly precipitated by the addition of ethanol and then collected through centrifuge. After residual cyclohexane and ethanol evaporated at room temperature, the obtained nanocrystals were dispersed again in chloroform (1 mL). Then, the above solution, SYLGARD silicone elastomer 184 (5 g) and curing agent (0.5g, Dow Corning) were mixed and stirred for 10 min. Finally, to remove chloroform and bubbles in the mixture, the luminescent ink was kept under vacuum condition for 1 h at room temperature. Here, the relative high concentration of nanocrystals in PDMS matrix was used to generate brighter downshifting luminescence.

**Preparation of aqueous lanthanide nanocrystals**

Firstly, 500 µL of lanthanide nanocrystals dispersion in chloroform (0.05 mmol) is mixed with 5 mL of chloroform and 5 mL of DSPE-PEG2000-CH3 solution (25 mg/mL in chloroform) in a 100 mL pear-shaped flask. Under heating in a 39°C water bath, the chloroform in the flask is rapidly evaporated by rotary evaporation until a transparent thin film is visible at the bottom of the flask. The flask is then vacuum dried for 60 minutes. Immediately after, 4 mL of deionized water heated to 100°C is added to the flask, and the mixture is sonicated at 100°C to promote the dispersion of the thin film. Finally, the colloidal dispersion is transferred to a centrifuge tube and centrifuged at 20,000 rpm at 10°C for 25 minutes, after which the supernatant is removed and the pellet is resuspended in 1 mL of deionized water. It should be noted that all aqueous nanoparticles need to be filtered through a 0.22 μm syringe filter before being used for in-vivo imaging.

***In-vivo* imaging**

Firstly, one Balb/c mouse was anesthetized by intraperitoneal injection of anesthesia (2% Avertin solution, v/v, 10 μL/g body weight) and carefully shaved to remove all body hair. Then, 200 μL of the prepared water-dispersed NaYF4: 50%Er@ NaYF4 (0.015 mmol) was orally administrated into the anesthetized mouse and a non- anesthetized one. The intestinal peristalsis of the two mice was imaged using an InGaAs CCD camera under laser excitation at 980 nm, in which 1100 nm and 1400 nm long-pass filters (FELH1100 and FELH1400, Thorlabs, Inc.) were used.

**Structure characterization**

Transmission electron microscopy was performed on HT-7800 transmission electron microscope with an accelerating voltage of 120 kV. The photoluminescence emission spectra were recorded on Edinburgh Instrument FLS980 Spectrometer (InAsGa PIN detector: 900-1700 nm) equipped with the external 980 nm (Changchun New Industries Optoelectronics Tech. Co. Ltd.). The NIR luminescent imaging system (Shanghai United Digital Biotech. Co. Ltd., NIR-II-ST) was equipped with a commercial InGaAs array detector (Princeton Instruments, NIRvana 640, 1000-1700 nm). X-ray diffraction (XRD) analysis was carried out on a Bruker D8 diffractometer with the Cu Kα line at 40 kV and 20 mA.


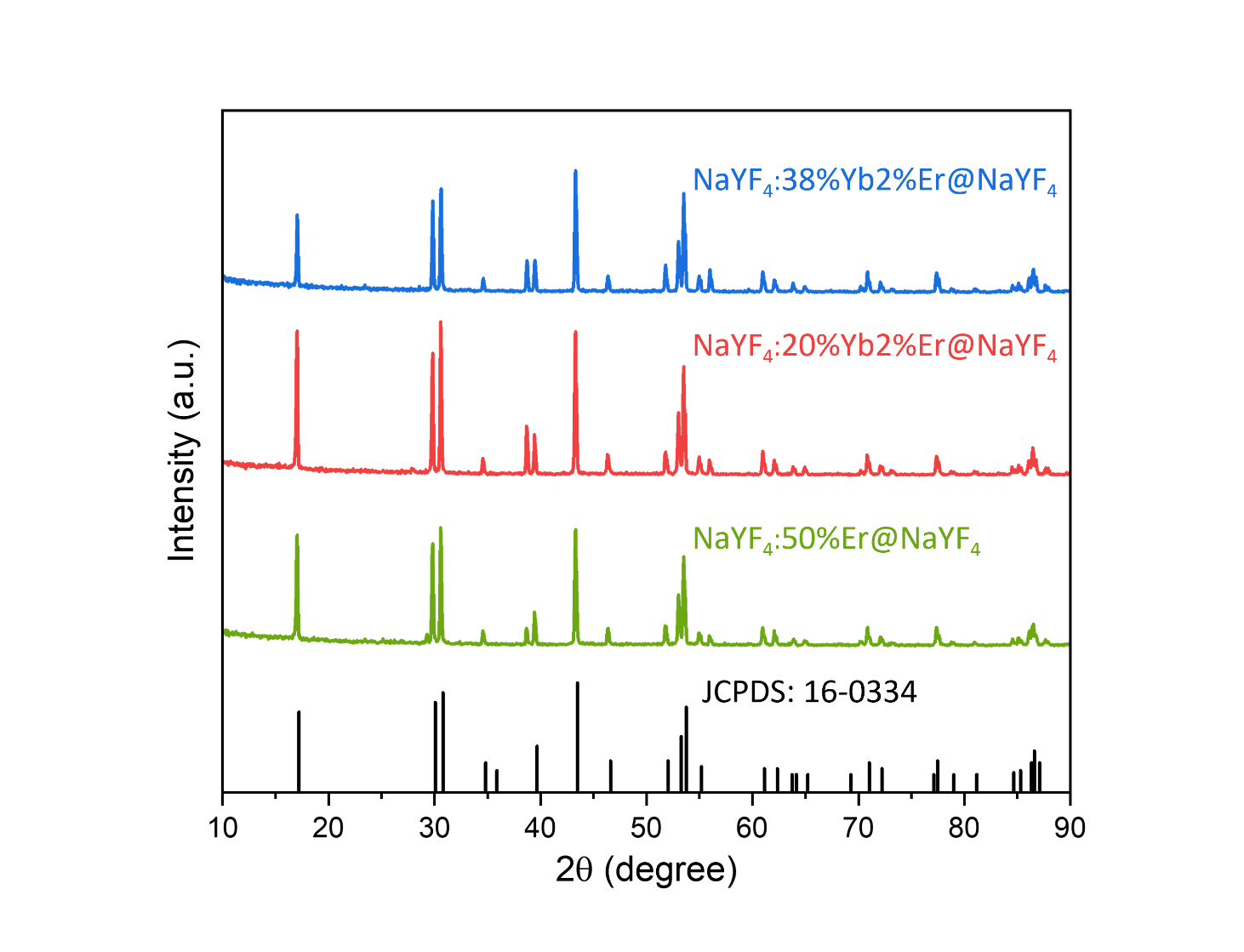


**Fig. s5** X-ray diffraction (XRD) patterns of the as-synthesized nanocrystals.


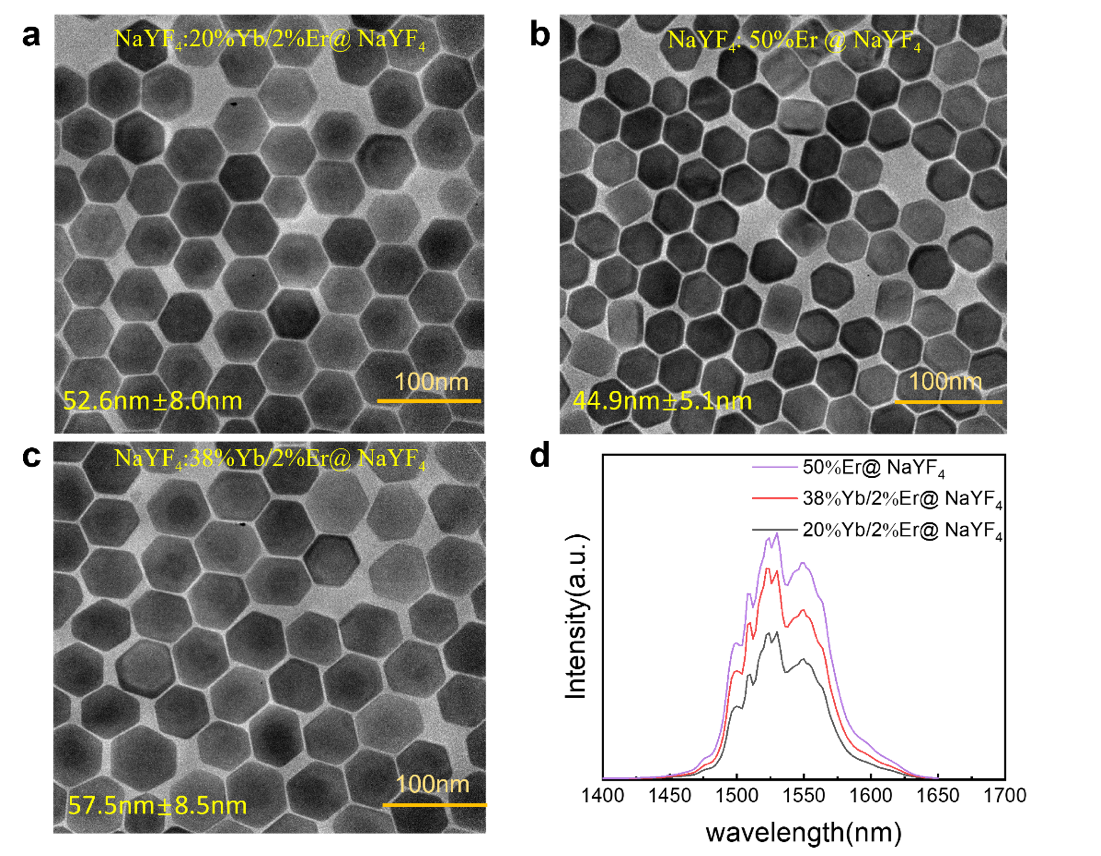


**Fig. s6** Characterization of NaYF4: different Er2+ content with different Yb2+ content.(a-c) TEM images of NaYF4:20%Yb/2%Er@NaYF4, NaYF4:50%Er@NaYF4 and NaYF4:38%Yb/ 2%Er@NaYF4. (d) Under 980 nm excitation, luminescence emission spectra of NaYF4:20%Yb/2%Er@NaYF4, NaYF4: 50%Er@NaYF4 and NaYF4:38%Yb/2%Er@NaYF4 in the spectral range of 1400-1700 nm.


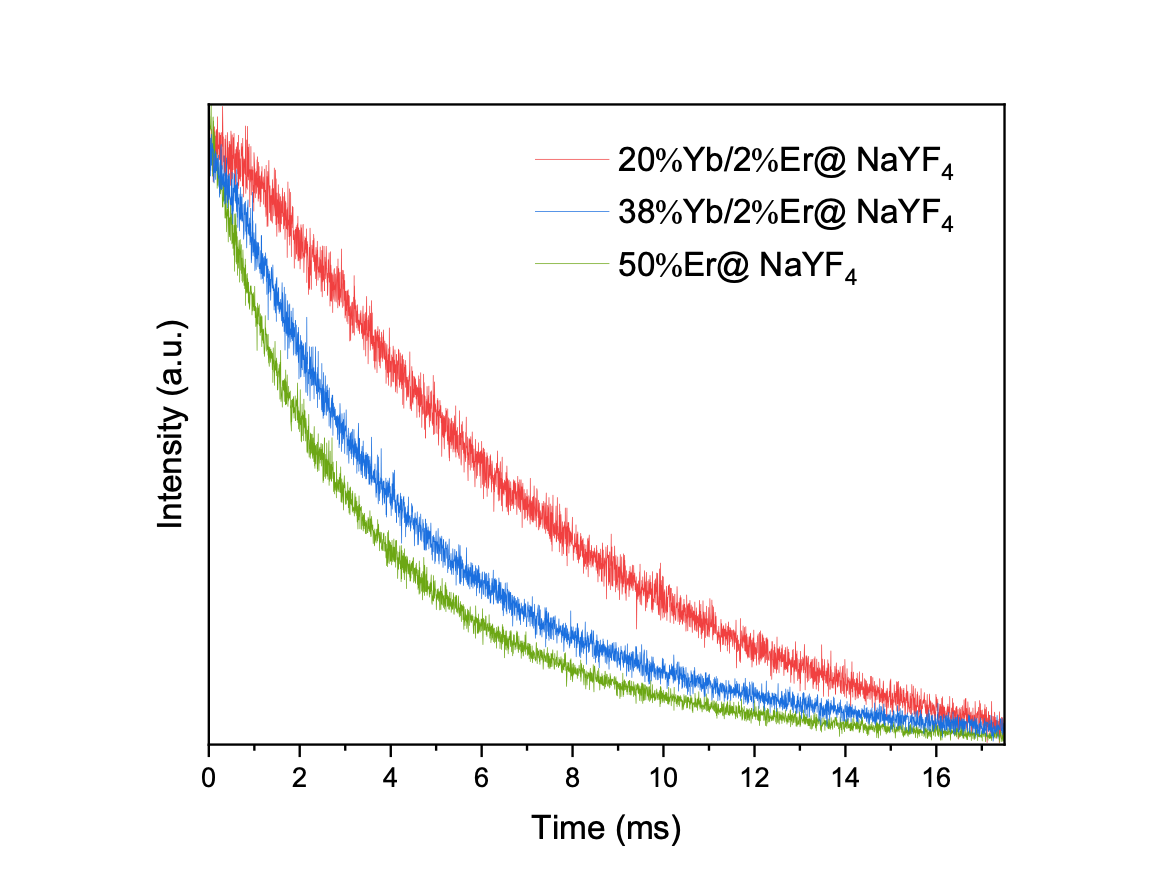


**Fig. s7** The luminescence decay curves at 1525 nm versus different samples (NaYF4:20%Yb/2%Er@NaYF4, NaYF4: 50%Er@NaYF4 and NaYF4:38%Yb/2%Er@NaYF4) under the 980 nm pulsed laser excitation.

**Supplementary Note 6 The details of the DPNet network**

In our experiments, we specifically constructed the training dataset of the end-to-end Reconstruction Network (DPNet) for the NIR-COFI system as follows: First, video materials exhibiting spatiotemporal sparsity were curated from public platforms and uniformly sampled to obtain 20-frame instantaneous image sequences per clip. In parallel, static images (e.g., DAVIS2017) from public repositories were used to synthesize 20-frame dynamic sequences by simulating translational/rotational/zoom motions, local deformations, and both global and local intensity variations. Together, these procedures yielded approximately 1,000 dynamic scenes, denoted for the *i*-th sample as . For each sample, a set of aperture spatially aligned with was provided, and the compressed observation was generated according to the snapshot compressed imaging forward model: per-pixel multiplication followed by summation across frames,

after which random noise was added to obtain the network input

Each training sample is therefore a triplet (,,), where serves as ground truth, is the known coding mask, and is the corresponding measurement. The sizes and preprocessing (including normalization and noise statistics) are kept consistent between training and testing, resulting in a physically faithful, high-quality dataset that covers diverse motion patterns and brightness changes.


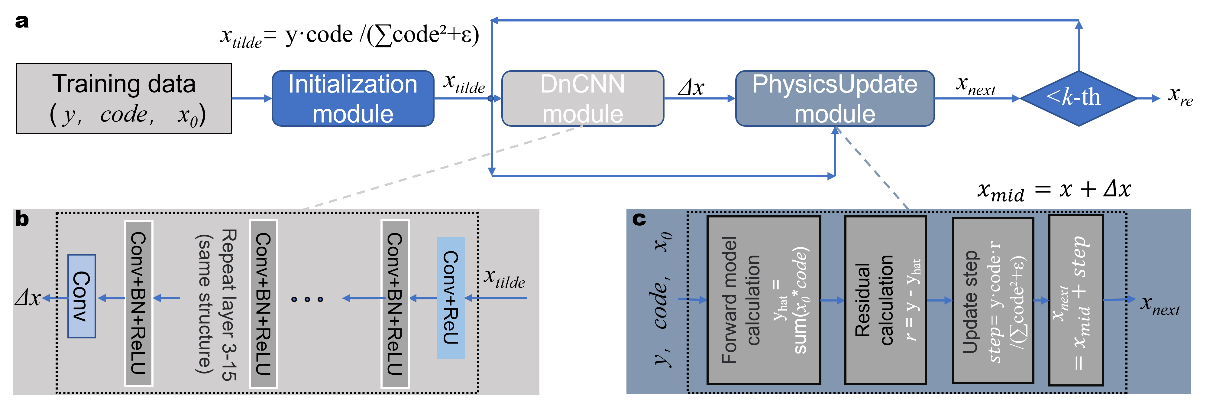


**Fig. s8** The flowchart of DPNet. BN: Batch normalization, ReLU: Rectified linear unit, Conv: Convolutional layer.

The architecture of the proposed DPNet is illustrated in **Fig. S8**. For each pixel, an analytical initialization is derived as

which yields the initial estimate, This expression corresponds to the minimum-norm analytical solution of the underdetermined linear system , with a small stabilizing term ε. The triplet (,,) as the input of the network, DPNet achieves the mapping from *y* to *x0* through the cascaded learning of *K* stages, enabling the inference process to only require the input of the *y* to obtain an approximate solution for *x0*and apply it for subsequent processing.

Each stage comprises two sequential modules: a DnCNN-based residual module and a Physics-Update module, described as follows.

**(1) DnCNN Module — Residual Feature Refinement.**
This module learns to refine the current estimate by predicting the residual between the network input and the ground truth. The concatenated input [ *y*, *code*, *xcur*] is processed by a lightweight DnCNN-style convolutional block (*xcur* is reconstruction results of previous one stage). It consists of an initial 3×3 convolution + ReLU (Rectified linear unit) layer, followed by depth-2 identical Conv-BN (Batch normalization)-ReLU blocks, optionally equipped with squeeze-and-excitation (SE) attention. The final 3×3 convolution maps the features to C channels. A residual connection produces the intermediate output .This design effectively captures high-frequency corrections while keeping the parameter count and GPU memory usage low, allowing efficient training on high-resolution data.

**(2) Physics-Update Module — Data-Consistency Enforcement.**
Given the current estimate X, the coding mask code, and the measurement y, the predicted measurement is computed. The residual is then projected back to the multi-channel domain via:

where ⊙ denotes element-wise multiplication, and the denominator prevents numerical instability in low-energy regions. The step size *γ* is implemented as a learnable parameter enabling adaptive control of the data-consistency strength for each stage. This operation ensures that the network output remains physically consistent with the forward model, leading to more stable convergence and interpretable gradient directions.

The entire forward propagation can be formulated as follows:
starting from：,

,

,

, for k=1,…,*K*.

The final output is regarded as the reconstructed result *xre*.

To guarantee physically guided convergence, a joint loss function is employed:

where the physical term evaluates pixel-wise consistency with the measurement model:

with denoting valid pixels.
The total loss is the sum of an L1 reconstruction term and a linearly weighted physical term, controlled by the parameter *λ*. A warm-up strategy gradually increases *λ* during early epochs to prevent unstable training caused by excessive physical constraints at initialization.

By integrating an analytical prior initialization with a DnCNN-based residual prior, DPNet establishes a hybrid 'prior + data-consistency cascade.' Crucially, it transcends pure end-to-end learning by employing a Physics-Update Module, which iteratively corrects reconstructions based on measurement residuals (*r = y - yhat*) to strictly adhere to the optical model . This strategy not only leverages the interpretability of traditional algorithms and the flexibility of deep learning to guide convergence but also prevents hallucinated features by anchoring the output to physical constraints.

**References**

1. Horton, N. G. et al. In vivo three-photon microscopy of subcortical structures within an intact mouse brain. *Nat. Photon.* **7**, 205-209 (2013).
2. Zubkovs, V. et al. Spinning-disc confocal microscopy in the second near-infrared window. *Sci. Rep.* **8**, 13770 (2018).
3. Wang, F. et al. Light-sheet microscopy in the near-infrared II window. *Nat. Methods* **16**, 545–552 (2019).
4. Wang, F. et al. In vivo NIR-II structured-illumination light-sheet microscopy. *Proc. Natl. Acad. Sci. U.S.A.* **118**, e2023888118 (2021).
5. Teledyne Princeton Instruments. NIRvana 640 SWIR Camera. <https://www.princetoninstruments.com/products/nirvana-swir-cameras/nirvana-640> (accessed Jan 2025).
6. Culley, S. et al. Quantitative mapping and minimization of super-resolution optical imaging artifacts. *Nat. Methods.* **15**, 263-266 (2018).
7. Zhang, H. et al. Tm³⁺-Sensitized NIR-II Fluorescent Nanocrystals for In Vivo Information Storage and Decoding. *Angew. Chem. Int. Ed.* **58**, 10153-10157 (2019).
